# Biomaterials to Neuroprotect the Stroke Brain: A Large Opportunity for Narrow Time Windows

**DOI:** 10.3390/cells9051074

**Published:** 2020-04-26

**Authors:** Daniel González-Nieto, Rocío Fernández-Serra, José Pérez-Rigueiro, Fivos Panetsos, Ricardo Martinez-Murillo, Gustavo V. Guinea

**Affiliations:** 1 Center for Biomedical Technology, Universidad Politécnica de Madrid, 28040 Madrid, Spain; rocio.fernandez@ctb.upm.es (R.F.-S.); jose.perez@ctb.upm.es (J.P.-R.); gustavovictor.guinea@ctb.upm.es (G.V.G.); 2 Departamento de Tecnología Fotónica y Bioingeniería, ETSI Telecomunicaciones, Universidad Politécnica de Madrid, 28040 Madrid, Spain; 3 Biomedical Research Networking Center in Bioengineering Biomaterials and Nanomedicine (CIBER-BBN), 28029 Madrid, Spain; 4 Departamento de Ciencia de Materiales, ETSI Caminos, Canales y Puertos, Universidad Politécnica de Madrid, 28040 Madrid, Spain; 5 Neurocomputing and Neurorobotics Research Group: Faculty of Biology and Faculty of Optics, Universidad Complutense de Madrid, 28040 Madrid, Spain; fivos@ucm.es; 6 Brain Plasticity Group, Health Research Institute of the Hospital Clínico San Carlos (IdISSC), 28040 Madrid, Spain; 7 Department of Translational Neuroscience, Instituto Cajal (CSIC), 28002 Madrid, Spain; r.martinez@cajal.csic.es

**Keywords:** stroke, brain ischemia, inflammation, excitotoxicity, oxidative stress, spreading depression, neuroprotection, drug delivery, biomaterials, polymers, nanoparticles, hydrogels

## Abstract

Ischemic stroke represents one of the most prevalent pathologies in humans and is a leading cause of death and disability. Anti-thrombolytic therapy with tissue plasminogen activator (t-PA) and surgical thrombectomy are the primary treatments to recanalize occluded vessels and normalize the blood flow in ischemic and peri-ischemic regions. A large majority of stroke patients are refractory to treatment or are not eligible due to the narrow time window of therapeutic efficacy. In recent decades, we have significantly increased our knowledge of the molecular and cellular mechanisms that inexorably lead to progressive damage in infarcted and peri-lesional brain areas. As a result, promising neuroprotective targets have been identified and exploited in several stroke models. However, these considerable advances have been unsuccessful in clinical contexts. This lack of clinical translatability and the emerging use of biomaterials in different biomedical disciplines have contributed to developing a new class of biomaterial-based systems for the better control of drug delivery in cerebral disorders. These systems are based on specific polymer formulations structured in nanoparticles and hydrogels that can be administered through different routes and, in general, bring the concentrations of drugs to therapeutic levels for prolonged times. In this review, we first provide the general context of the molecular and cellular mechanisms impaired by cerebral ischemia, highlighting the role of excitotoxicity, inflammation, oxidative stress, and depolarization waves as the main pathways and targets to promote neuroprotection avoiding neuronal dysfunction. In the second part, we discuss the versatile role played by distinct biomaterials and formats to support the sustained administration of particular compounds to neuroprotect the cerebral tissue at risk of damage.

## 1. Introduction

Demographic change is an undeniable reality in modern countries. In the coming decades, an increasing number of pathologies are expected to occur as a consequence of aging. Aging and additional risk factors, such as hypertension, cholesterol, obesity, and sedentary lifestyle, will contribute to an increase in the prevalence of pathologies caused by brain, heart, and arterial dysfunctions. The sudden occlusion of cerebral arteries produces brain ischemia. This fatal disease is a leading cause of death and disability among adults, comprising ~85% of all stroke cases in comparison with hemorrhagic strokes (~15%), which are caused by an arterial rupture. Stroke continues being a devastating disorder, with mortality rates of 30% and 50% at 1 month and one year, respectively, after the initial attack [1]. The reversibility and duration of occlusion are the first determinants of the extent of damage influencing a patient’s prognosis. A main cause of stroke is the occlusion of the middle cerebral artery, which supplies oxygen and nutrients to sensory and motor areas. Occlusion of this artery is commonly associated with contralateral motor and sensory dysfunction, but, depending on the specific occlusion and affected artery/s, other clinical symptoms might appear, such as cognitive and perceptual deficits with varying degrees of affectation. Preventative programs to reduce risk factors have diminished the burdens of this disease.

Acute therapies for ischemic stroke are based on the re-canalization of occluded vessels through pharmacologic and invasive surgical procedures. In contrast, during the chronic stage, physical and cognitive rehabilitation therapies might work in a minority of patients, especially in subjects with less extensive damage after the initial insult [2,3]. It is clinically accepted that the administration of a tissue plasminogen activator (t-PA) for clot dissolution—alone or in combination with surgical procedures such as endovascular thrombectomy for clot retrieval—constitute the most acceptable treatment to treat stroke patients in the early stages (acute phase). Although its efficacy has been demonstrated in clinical trials [4,5], the number of patients benefited by this procedure is unfortunately low, around 5% of all stroke patients [1,6], a fact ascribed to the narrow time window for t-PA administration (3–4.5 h after stroke) and because delayed thrombolytic therapy and blood reperfusion have been associated with a high risk of hemorrhagic transformation and oxidative stress, thus causing additional damage. Ischemic stroke produces a core of irreversibly damaged tissue surrounded by a salvageable area called the penumbra, which has a high risk of neuronal death following the initial infarct. Both the ischemic core and the penumbra area, if damaged, are generally responsible for the definitive lesion. Although most stroke patients show definitive lesion sizes 24–36 h after the onset of symptoms, in a third of patients, the final lesion size occurs after one week [7]. Thus, the definitive area of injury depends of both the time of blood flow occlusion/oxygen deprivation (primary initial damage) and the so-called secondary injury that will affect the peri-lesional penumbra and non-damaged areas, transforming them in irreversibly damaged regions [8,9]. Many molecular and cellular events have been related to this secondary wave of damage, including, but not limited to: i) excitotoxicity mediated by uncontrolled release of neurotransmitters such as adenosine and glutamate concurrently with an overload of intracellular calcium [10], and ii) impaired mitochondrial functions and oxidative stress caused by free radicals and reactive oxygen/nitrogen species [11]. In addition, inflammation is a component of the pathophysiology of the brain in stroke, contributing to neuropil damage. Inflammation is mediated by microglia and the recruitment and infiltration from the blood to the brain of leukocytes that release pro-inflammatory and pro-apoptotic molecules [12]. Another contributor of secondary damage is the spreading depolarization and their associated inverse hemodynamic changes with the hypoperfusion of peri-lesional areas [13].

### 1.1. Lost in Clinical Translation

There is a narrow time window for therapeutic interventions based on recanalization procedures and attenuators of secondary damage to prevent neuronal death and damage. In the last few decades, intensive collaborative work has been done to identify the precise targets involved in secondary damage whose modulation can be exploited to neuroprotect the brain within this narrow time window. Although many of these targets have been very promising at the preclinical level, there has been an undeniable lack of translatability into clinics. It has been estimated that more than 1000 therapeutic molecules have been tested in the preclinical phase. About ten percent of these molecules entered clinical trials; however, except for some trials [14,15], all of them failed to present positive outcomes [16]. Several arguments have been considered to explain this desolate scenario, such as a lack of adequate animal models, a lack of consensus between preclinical studies, an oversimplification of the pathogenic molecular and cellular routes and pathways, the heterogeneity of clinical trials, and the poor standardization of clinical procedures. Specifically, there has been a lack of connections between preclinical and clinical trials. For example, neuroprotective compounds that were tested successfully in transient ischemic models were used later in patients with permanent ischemia because recanalization approaches only benefit a minority of subjects [17]. These biomolecules were assayed in healthy young animals submitted to stroke, while brain ischemia mostly affects aged populations with previous comorbidities [17]. Even assuming similar neuroprotective pathways and targets between human and rodents, the blood-brain barrier permeability, concentration ranges for drug effectiveness, and molecular clearance by the glymphatic system are, with a strong probability, different between species [18]. For example, the endothelial cell thickness of the blood-brain barrier oscillates from ~200 nm in mice to ~400 nm in human brain tissue, which probably influences the permeation of molecules differently between both species [19,20]. A renewal of the total cerebrospinal fluid is produced in humans every 5 h, while in the rodent brain, this process occurs more rapidly (every hour) [21]. These values likely produce differences in the clearance rates of biomolecules. Even when we consider only the same rodent species, there are examples throughout the literature where similar stroke models have caused variable damage and affectations between different strains [22]. Particular drugs can even exert both neuroprotective and non-neuroprotective effects depending on the rodents and strains used [23]. Due to this great variability in therapeutic effects and their efficacy, even between the closest mammalian species, it is not surprising that there is difficulty in establishing therapeutic connections between species, including humans.

### 1.2. The Blood-Brain Barrier

While we advance in resolving of these issues to identify promising targets and drugs, and to establish better stroke models (i.e., human organoids for drug screening), it is also a priority to progress the development of efficient systems for pharmacological administration into central nervous tissue, especially designed for humans. Non-invasive (systemic) and invasive (intracerebral) routes of administration have been preclinically and clinically explored. The main handicap of the systemic route is the inability of many drugs and biomolecules to cross the blood–brain barrier (BBB) and reach the brain with efficacy [24]. The restrictive permeability of the BBB and, in particular, the abundance of tight junctions that encircle endothelial cells, complicates the entrance of biomolecules into the brain by crossing the luminal and antiluminal lipid membranes that face the blood lumen and the brain parenchyma, respectively. Under physiological conditions, several highly regulated mechanisms have been described to facilitate the passage of specific molecules through this barrier. These mechanisms include transporter proteins, transcytosis processes, simple diffusion, and paracellular transport in the tight junction borders between adjacent endothelial cells [25]. In general, the active mechanisms of transport permit the entrance of polar molecules, such as glucose and amino acids, while small hydrophobic substances generally cross the BBB by simple diffusion [26]. In addition, some large molecules can physiologically pass through transcytosis mechanisms. Under pathological conditions, the scenario is different, since the permeability of the BBB is impaired very early after injury [27]. For example, in ischemic rats, the permeability of the BBB is compromised as soon as 2–3 h after occlusion [28]. Despite the BBB being substantially impaired after injury, in most systemic applications, it is unknown how much of the administered drug may effectively reach the brain and whether the brain’s local concentrations that are effective in rodent models are also valid for humans due to differences in drug clearance. All these facts obviate any control that we believe to have over drug kinetics at effective doses to target the salvage penumbra. An alternative route for systemic administration is intracerebral. Although this route is more invasive, an intracerebral injection offers significant advantages, e.g., the direct administration of drugs in the area/s of interest, although these drugs are also exposed to rapid degradation and clearance, which is a strong limitation if persistent pharmacological effects are needed.

For many neuroprotective agents, the amount of drug and time required to achieve the optimal therapeutic effect is not addressed in the majority of studies, causing additional bewilderment. After decades of study, the potential of many natural and artificial polymers to treat many diseases, including those of neural origin, has come to light. Different biomaterials and formats can be designed to have adequate biocompatibility with nervous tissue to sustain drug delivery, thus reducing the frequent administration of particular compounds with poor half-lives due to their greater susceptibility to the rapid degradation and decay of activity in the injured neurovascular microenvironment. Biomaterials might satisfy the therapeutic need for pharmacologic release, extending the action of drugs, at least during the time window of the salvage penumbra. In this review, we discuss relevant studies in the field that anticipate an exponential leap in the use of advanced biomaterials as micro- and nano-pumps to support the pharmacological delivery of neuroprotective compounds that maximize the duration and effectiveness of the limited therapeutic window of stroke patients.

## 2. Neuroprotective Strategies for Recovery after Ischemic Stroke

**Figure 1:**
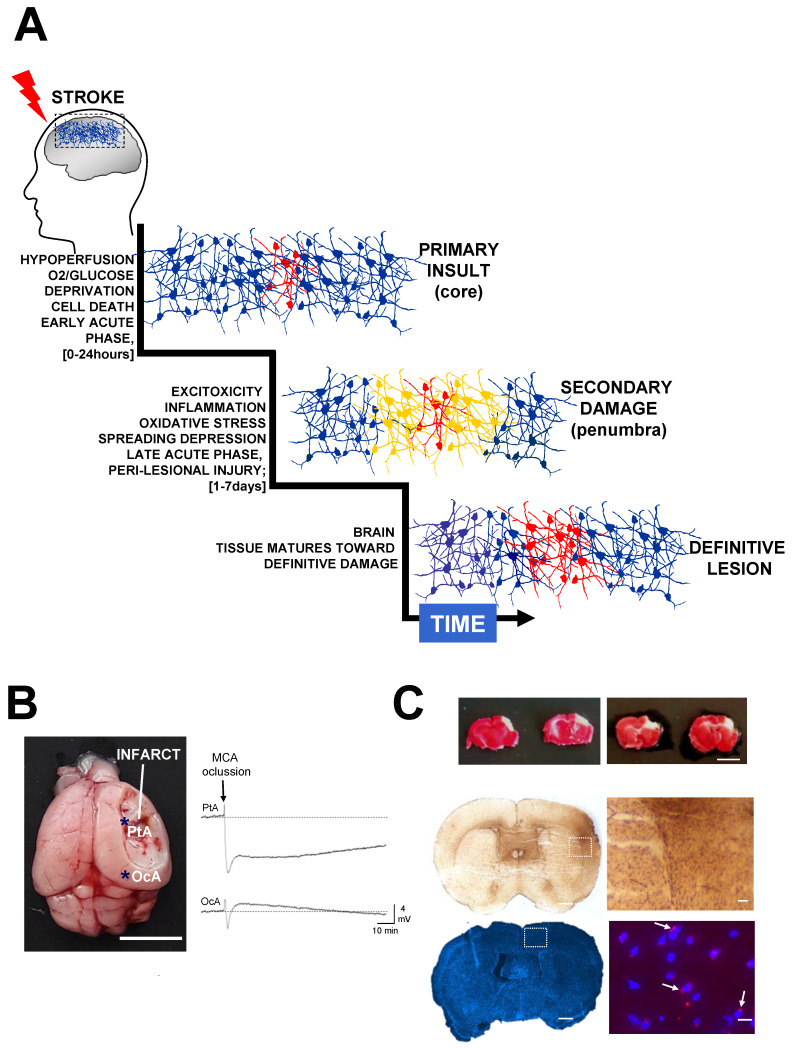
Secondary injury increases lesion extension after stroke. (**A**) The sudden occlusion of a brain artery causes hypoperfusion and oxygen deprivation, producing a core of irreversibly damaged tissue surrounded by a penumbral area at risk of being damaged. After hypoxia, stroke triggers excitotoxicity, blood flow changes, inflammation, and oxidative stress, which produce secondary damage, thus extending the area of the lesion. The relevant time phases post-stroke have been defined as acute, sub-acute and chronic [30,31]. During the acute phase (hyperacute ~0–24 h; late acute ~1–7 days) it is possible to therapeutically neuroprotect the brain, thereby preventing the detrimental effects of secondary damage. The definitive damage is relatively well established several days after stroke, when neuroprotective agents are no longer efficient. During sub-acute (~7 days–6 months) and chronic (>6 months) phases, tissue reorganization can take place via rehabilitation and neural repair strategies. (**B**) Stroke produces peri-infarct depolarizations that cause vasoconstriction and blood flow reduction, propagating hypoperfusion to peri-lesional areas and causing additional damage. The image shows a mouse brain (MCAO model, scale bar 0.5 cm) 24 h after permanent ischemia (MCA ligation). Immediately after MCA occlusion, a wave of terminal depolarization was electrophysiological recorded in peri-lesional areas (parietal cortex, PtA) bordering the infarct core (asterisk in the image). Note the existence of brief depolarization waves in non-damaged distant areas (occipital, OcA), which, in metabolically compromised ischemic tissues, also might cause intense vasoconstriction and hypoperfusion. (**C**) At the top, representative coronal brain sections from two mice stained with TTC (2,3,5-triphenyltetrazolium chloride) 24 h after MCA occlusion at distal level respect the Circle of Willis. In this specific stroke model, the infarct area (in a white colour) is mainly restricted to the cortex (scale bar 0.5 cm) [83,84,85]. In the middle, as part of the inflammatory response, an intense astrogliosis (Glial Fibrillary Acidic Protein staining) can usually be detected in the infarcted hemisphere in relation to the contralateral hemisphere (scale bars 700 μm and 100 μm, respectively). In the bottom, representative brain sections stained with dihydroethidium (DHE) to detect reactive oxygen species and intracellular superoxide. In this example, as early as 8 h after MCAO, the most intense fluorescence was detected in the peri-lesional tissue in perinuclear locations (the nuclei are stained with DAPI, scale bars 700 μm and 10 μm respectively).

Cell death after ischemia occurs rapidly in the regions that receive less blood flow. The duration of ischemia and the site of occlusion define the affected site and the initial amount of damage in the cerebral area. Oxygen deprivation causes Na^+^/K^+^-ATPase and membrane potential dysregulation. After the onset of ischemia, the extension of damage is largely determined by the progressive transformation of the salvaged penumbra into damaged tissue, with the ischemic penumbra representing a zone of viable tissue adjacent to the ischemic core that has a high risk of cell death. In the pathogenesis of stroke or in response to ischemia, different biochemical, molecular, and cellular signals, including apoptotic factors, emanate from the ischemic core, propagating cerebral damage towards the penumbra and surrounding initially non-damaged tissue. Taken together, death executors include abnormal excitability, inflammation, oxidative stress, and spreading depolarization (Figure 1).

The variability of lesions between patients is creating new controversies regarding the proper time windows for pharmacological and endovascular interventions [29]. In patients, the improvement of clinical signs and responses to treatment is greater for smaller ischemic lesions. Because the size of the damaged area, degree of affectation and recovery of ischemic patients depend on both blood flow/oxygen deprivation and secondary damage, neuroprotective strategies have been developed to control the events mostly responsible for this secondary damage during the acute phase of a stroke [30,31].

### 2.1. Excitotoxicity

Excitotoxicity occurs after a stroke due to the uncontrolled release of neural excitatory neurotransmitters. Correcting this dysregulation has been considered for decades as a potential approach for neuroprotection following stroke. Oxygen deprivation causes energy (ATP) exhaustion and the impairment of ionic gradients, especially intracellular potassium depletion and anoxic depolarization. Changes in resting membrane potential occur parallel to the massive increase of excitatory amino acid neurotransmitters in the extracellular space, which include adenosine and mostly glutamate. In addition, energy depletion impairs neurotransmitter re-uptake and clearance. The excessive activation of glutamatergic receptors (NMDA, AMPA, and kainate) is neurotoxic with concomitant entrance of large amounts of calcium, oxidative stress, mitochondrial dysfunction, and modifications in the expression of genes and the level of protein activation, thus inducing cell necrosis or apoptosis. For example, calcium overload increases intra-mitochondrial calcium levels, causing the opening of the mitochondrial permeability transition pore, which impairs the permselectivity of the mitochondrial barrier, releasing ions and distinct molecules to the cytosol, such as Cytochrome c, which triggers cellular apoptosis. Calcium overload secondary to activation of excitatory glutamate receptors boost anomalous increase of reactive oxygen and nitrogen species levels. Large amounts of nitric oxide (NO) generated by neuronal NO synthase are produced after the large-scale stimulation of the glutamate receptors. Excessive NO interacts with the superoxide anion (O^2−^) forming very toxic reactive oxygen species (ROS) molecules, such as peroxynitrites (ONOO^−^). In the presence of a variety of ROS, cellular proteins are vulnerable to damage by oxidative stress, which may take the form of nitration or oxidation of various aminoacid residues. Oxidative stress produces the oxidation/nitration of proteins and lipids and DNA fragmentation, leading to neuronal death. In addition, high levels of glutamate cause blood–brain barrier dysfunction and brain edema [32]. Thus, the excitotoxicity mediated by the binding of glutamate to NMDA and AMPA receptors is a major mechanism of secondary damage and neuronal death after stroke, NMDA receptors being mainly responsible for increasing calcium entrance and subsequent neurotoxicity.

Due to the transcendental importance of glutamate receptors in the pathogenic mechanisms of stroke, glutamate excitotoxicity has been mainly taken as a target to design effective glutamate receptor antagonists to prevent mitochondrial dysfunction and free radical generation. In this way, in models of transient ischemia in rats, NMDA receptor antagonists, such as MK-801, have shown neuroprotective effects by decreasing the infarct size and brain edema in parallel with an improvement of behavioral deficits [33]. NMDA receptor activation depends on, in addition to glutamate, glycine binding. Thus, glycine antagonists have been used successfully in preclinical studies [34,35]. As an example, the glycin antagonist GV150526A has proved efficacious in reducing the infarct volume and partially preserving the functionality of the cortical forepaw and hindpaw somatosensory areas [34]. In striking contrast, several drugs that hypoactivate NMDA receptor function have failed at the clinical level [36,37,38]. Apart from the heterogeneity of patients in clinical trials and the lack of preclinical/clinical connections, several interpretations have been drawn, including the very short time window for drug actions and the masking of therapeutic efficacy due to significant side effects. Inhibitors of glutamate receptors impair normal glutamate function in non-damaged networks, a fact likely ascribed to a lack of precision in targeting the peri-lesional penumbra. In addition, the complexity of nature surpasses our more simplistic expectations. Two different types of NDMA receptors have been defined in relation to its cellular location. Synaptic NMDA receptors promote cell survival through ERK/CREB activation and BDNF production, while the activation of NMDA receptors that are localized in non-synaptic areas leads to pro-apoptotic events and the suppression of survival signals [37]. Thus, the direct targeting of NMDA receptors might simultaneously produce opposite effects, pro-survival and pro-death signals. These pro-death signals might contribute to masking the positive effects of excitoxicity inhibition. Recent therapeutic approaches have been based on the inhibition of downstream pathways instead of the direct blocking of NMDA receptors. These strategies have been specifically focused on suppressing the death signals from pro-apoptotic extra-synaptic NMDA receptors, which are enriched, unlike the synaptic NMDA receptors, with GluN2B subunits. Targeting the triad GluN2B-PSD95-nNOS pathway represents a very attractive strategy, since nNOS overactivation leads to increasing levels of NO, free radicals, and peroxynitrite. Different interfering peptides against PSD95 or nNOS have been used with promising results, as was the case for the NA-1 peptide that dissociates NDMA receptors from PSD95 subunits, reducing the infarct size and improving functional outcomes after stroke in rodents and macaques [39,40]. In clinical trials, the NA-1 peptide has also shown significant benefits by reducing the appearance of new lesions in stroke patients [41]. Recently, in a multicentre trial, the NA-1 peptide produced a significant improvement among stroke patients that did not receive t-PA, while unexpectedly, this PSD95 inhibitor did not show favorable outcomes in t-PA-treated stroke patients [14].

### 2.2. Oxidative Stress

Free radical generation and oxidative stress are pathological phenomena tightly linked to excitotoxicity. The abnormal rise of intracellular calcium levels upon the abnormal release of excitatory neurotransmitters leads to oxidative damage. Oxidative stress usually results from excessive ROS production, mitochondrial dysfunction, failure of anti-oxidant mechanisms, or a combination of these factors [42]. Oxidative stress plays an essential role in the pathogenesis of cerebral ischemia-reperfusion (I/R) injury [43,44] and has a critical responsibility in the pathogenesis of post-stroke neural damage by inducing neuronal death and apoptosis, severely impairing neurological function [45]. A main source of ROS is the mitochondria. Most mitochocondrial and non-mitochocondrial ROS are able to cross the cellular membrane towards the extracellular compartment, this spreading the pathology to neighbour cells. After ischemia/reperfusion, reactive nitrogen and oxygen species (RNOS) are overproduced, exceeding the capability of cellular systems for RNOS clearance. RNOS overproduction after reperfusion plays an important role in the pathogenesis mechanisms of secondary damage, although permanent ischemia with no recanalization might also induce the production of different free radicals, including O^2−^ [46]. As stated above, free radicals produce lipid peroxidation, protein oxidation and denaturation, enzyme inactivation, protein aggregation, damage of cellular membranes, DNA fragmentation, additional level of intracellular calcium by its release from intracellular organelles, damage to the cytoskeleton, cerebral edema, and the breakdown of BBB, mostly because ROS activate different matrix metalloproteinases [10,47]. In addition, ROS enhance central and peripheral inflammation. It is widely accepted that free radicals and ROS contribute to extend the area of damage affecting different cell populations, including neurons, astrocytes, oligodendrocytes and endothelial cells.

Oxidative stress and hydroxyl radical formation can be prevented by complex scavenger systems that are present in the cytoplasm and different cellular organelles (mitochondria). Several enzymes such as superoxide dismutase (SOD), glutathione peroxidase and catalase; and non-enzymatic natural compounds such as ascorbate, vitamin E, and glutathione, show anti-oxidant properties. For example, SOD converts O^2−^ in H_2_O_2_, which can later be detoxified by catalase and glutathione peroxidase, producing O_2_ and H_2_O. This detoxification route is important because H_2_O_2_ can produce hydroxyl radicals through a Fenton reaction mechanism involving Fe^2+^/F^3+^ species, inducing serious cellular injury. In general terms, the experimental treatment with these antioxidant enzymes reduces the infarct size after stroke. It has been shown that transgenic mice overexpressing SOD had less extensive damage after cortical ischemia, as well as increasing levels of the anti-oxidants glutathione and ascorbate in the non-damaged surrounding tissue [48]. In a model of transient ischemia in rats, treatment with SOD and catalase conjugated with polyethylene glycol (PEG) increased by four hundred times the half-life of both enzymes in circulation and reduced the total infarct volume [49]. Edavarone, a very powerful antioxidant agent, has been used both pre- and clinically. This compound increases the activity of SOD, catalase, and glutathione peroxidase for O^2−^ and H_2_O_2_ detoxification, as well as modifies the NO content [50]. The volatile intercellular messenger NO exerts a close relationship with stroke contributing to brain damage. The toxic effect of NO closely associated with excitotoxicity, is related to its production through inducible or neuronal NO synthase (iNOS; nNOs, respectively). Noxious NO is involved in the production of nitrates and the release of deleterious free radicals. Edavarone has a strong scavenger potential, capturing H_2_O_2_ -derived hydroxyl radicals and increasing the activity of endothelial eNOS, while it downregulates the levels of iNOS and nNOS; thereby decreasing NO and ONOO^−^ levels [50]. Edavarone has been associated with a reduction of the infarction size, secondary edema and BBB dysfunction, and is clinically used in Japan to treat brain ischemia in patients [15]. Targeting key steps in the cascade of I/R, including lowering NO production has been also proposed in our laboratory [45]. Other antioxidant compounds have been assayed with less success at the clinical level. This applies, for example, to the free radical scavenger, nitrone NXY-059, an agent that, when administrated 3 h after I/R (but not 6 h later), produced smaller infarctions in stroke rats [51]. This therapeutic efficacy was corroborated in primates, where NXY-059 administrated 4 h after ischemia/reperfusion reduced the infarct volume in parallel to behavioral improvement [52]. However, the proof of concept of the therapeutic potential of NXY-059 extracted from animal models of the disease did not translate into clinical improvements in stroke patients [53]. Other promising scavenging molecules, such as uric acid, have also failed to exert significant neuroprotection in clinical trials [54]. Except for edavarone, no clear evidence of the efficacy of other antioxidant compounds has been obtained to treat human stroke patients.

### 2.3. Inflammatory Response after Stroke

Inflammation is a central component in the pathophysiology of stroke. Preconditioning, a term related to exposure to low-dose lipopolysaccharide (LPS) before cerebral ischemia, was found neuroprotective in stroke models [55]. After stroke, central and peripheral inflammation is produced in the peri-lesional and lesional regions. As previously commented, central inflammation is mostly mediated by microglia, an immune brain-resident cell population of hematopoietic origin that becomes activated after brain injury. Microglial activation is produced rapidly, minutes to hours after brain ischemia, and becomes active for days or even weeks after a stroke [56,57]. Microglia activation leads to the production of a wide spectrum of inflammatory molecules and reactive oxygen species, some of which comprise BBB permeability, favoring peripheral blood cell infiltration into the brain.

#### 2.3.1. Central Inflammation

After brain injury, the microglia experience a morphological transformation, extending their processes toward the sites of damage. Then, the proliferation and phagocytic activity of the microglia is increased, regulating the inflammatory response via the secretion of pro- and anti-inflammatory factors. Similar to other hematopoitic-derived cells, such as macrophages, microglia cells function as immune mononuclear plastic phagocytic cells that become activated in response to injury and repair. Microglial activation in the central nervous system can be polarized towards two main opposite phenotypes, M1 and M2, which show pro- and anti-inflammatory roles [58]. Thus, depending on the phenotypes activated, microglia can produce either cytotoxic or neuroprotective effects. M1 microglia prevails at the end stage of disease at the injury site, just when the action reparative process of M2 microglia is dampened.

M1/M2 polarization phenotypes can be considered targets for stroke therapy. The discovery of new drugs related to M1/M2 polarization has enabled the realization of targeted therapies [59]. The M1 phenotype can release inflammatory mediators, such as tumor necrosis factor-alpha (TNF-alpha), interleukin-1Beta (IL-1-Beta), interleukin-6 (IL-6), metalloproteinases (MMP), iNOS, and NO, thus promoting oxidative stress via peroxinitrite, superoxide, and H_2_O_2_. These inflammatory mediators increase BBB permeability and promote the recruitment and infiltration of peripheral immune cells. M1 microglia and ROS lead to the activation of several matrix metalloproteinases, a family of endopeptidases that act outside cells and have pathological roles in the evolution of brain damage, including excitotoxicity, neuronal death, degradation of extracellular matrix (ECM) proteins, activation of different growth factors and cytokines, and cleavage of cell surface receptors and cell-to-cell adhesion proteins [60]. After brain ischemia, increasing levels of MMP-2 and MMP-9 in the infarct and peri-infarct areas degrade the ECM, thereby disrupting the endothelium and causing the BBB to open, as well as brain edema and hemorrhagic transformation. After stroke, MMP-9 deficient mice were associated with less damage and a better preservation of BBB integrity [61,62], and MPP-9 inhibition was translated into neuroprotection after stroke [63]. In contrast, polarization to the M2 microglia phenotype produces anti-inflammatory molecules, such as interleukin-4 and interleukin-13, facilitating both the clearance of toxic products and wound healing in collaboration with astrocytes. During development and in the adult, astrocytes play a role regulating brain morphology and function. Astrocytes help to maintain neuronal homeostasis and repair the BBB, as well as stimulate neovascularization. After brain damage, reactive astrocytes (astrogliosis) extend their processes around the site injury, creating a barrier (glial scar) to isolate and compartmentalize the damaged/ inflammatory tissue, thereby preventing the unaffected brain from being exposed to harmful signals, although scar formation after injury leaves permanent deficits in central nervous system (CNS) diseases [64]. Stroke provokes the loss of function of resident astrocyte glutamate transporters, mainly in the synaptic cleft, which leads to impaired astrocytic glutamate uptake reducing their ability to maintain low levels of extracellular glutamate [65]. The glial scar imposes a physical and chemical barrier for neural cell infiltration and axonal re-growth in the stroke cavity, avoiding functional rewiring and recovery after injury. The classical pro-/anti-inflammatory M1/M2 phenotypic classification, extracted from in vitro studies of microglial activation, is likely more complex, and additional phenotypes might coexist, as suggested by transcriptome and proteome analyses [66]. Several strategies have been developed to modulate microglia polarization and activation, as well as to regulate the expression, release, and effects of different cytokines. For example, microglia polarization towards the anti-inflammatory phenotype has been reported to ameliorate cerebral damage after ischemia [67,68].

#### 2.3.2. Peripheral Cell Infiltration and Inflammation

As commented above, peripheral myeloid cells, mostly neutrophils and monocytes/macrophages, are recruited after a stroke and transmigrate across the cerebral endothelium, crossing the BBB and infiltrating in peri-lesional and lesional areas. Microglia-derived MMPs play significant roles in disrupting the BBB, promoting leuckocyte infiltration. Peripheral cells produce different inflammatory molecules and factors that influence infarct evolution during early stages and remodel the extracellular matrix, producing structural/functional plasticity during the chronic stages. Neutrophils have shown both deleterious and neuroprotective effects. Harmful neutrophils cause the additional disruption of BBB, cerebral edema, hemorrhagic transformation, and damage due to the releasing of ROS, as well as different proteases, including MMPs, elastase, cathepsin G or proteinase 3, and inflammatory cytokines such as TNF-alpha, IL-1Beta, IL-6, which have been related to rapid neurotoxic effects [69]. The pro-inflammatory role of TNF-alpha remains controversial. For example, in a rat stroke model of ischemia-reperfusion, antibodies against TNF-alpha reduced the infarction size, leading to better neurological improvement [70]. Although these data support the neuroprotective benefits of inhibiting TNF-alpha, TNF or TNF-receptor deficient mice showed larger infarctions and worse outcomes than non-genetically modified mice, supporting the neuroprotective role of TNF in cerebral ischemia [71,72]. In contrast, inhibition of the IL-1 receptor produced less leukocyte infiltration from the peripheral blood to the brain, smaller infarct volumes, and better functional outcome [73]. Preclinical studies have shown that the inhibition of neutrophil infiltration enhances post-stroke functional recovery. For example, the inhibition of CXC receptors and neutrophil recruitment with Reparixin reduced brain damage, leading to functional improvement [74]. However, alternative studies have shown an opposite effect, finding that the inhibition of neutrophil activity and infiltration was not associated with functional improvement after stroke [75]. Similarly, monocytes/macrophages migrate to the injured brain, exerting both protective and detrimental effects. For example, in a model of ischemia reperfusion injury, C-C chemokine receptor type 2 (CCR2) deficient mice showed impaired monocyte and neutrophil chemoattraction and infiltration into the brain, with smaller infarction sizes, and reduced brain edema, which translated into neurological improvement [76]. In contrast, CCR2 inhibition with the antagonist INCB3344 was associated with poor monocyte infiltration that did not translate into functional post-stroke recovery [77]. The discrepancy between both studies might be related to the specific contexts of the analyses and the temporal window of CCR2 silencing; the pharmacological inhibition of CCR2 versus CCR2 genetic deficiency from development. These studies illustrate the complexity of monocyte infiltration and its involvement in the pathological evolution of brain damage after ischemia. Indeed, the infiltration of monocytes has been considered a positive signal for microglial activation reducing secondary damage and neuronal dysfunction. This was illustrated in a recent study, where C-X-C chemokine receptor type 4 (Cxcr4)-deficient stroke mice showed decreasing monocyte infiltration and increasing expression of microglia pro-inflammatory factors that translated into worse functional outcomes [78].

### 2.4. Spreading Depolarization

After brain ischemia, waves of depolarization (SD) initiate mostly on the border of the ischemic penumbra and propagate towards the peri-infarcted and non-damaged regions (Figure 1). These abrupt waves are accompanied by a loss of neuronal transmembrane ion gradients and impaired neurotransmitter release, energy metabolism failure, neuronal swelling, and dendritic beading. The occurrence of SD has been reported in other brain disorders, including migraine, traumatic brain injury, and subarachnoid and intracerebral haemorrhage. In non-metabolically compromised brain tissue (for example, in a migraine), these depolarization waves essentially cause hyperemia (increasing blood flow) as a normal hemodynamic response to recover ion gradients and energetic metabolism. However, in metabolically compromised tissue, like those after brain ischemia, SD causes intense vasoconstriction and blood flow reduction, preventing neuronal repolarization and propagating hypoperfusion to peri-lesional areas, producing additional damage [79]. This relative simplistic interpretation of hemodynamic changes in response to SD in healthy (hypermia) and non-healthy (hypoperfusion) brains is changing toward a more complex scenario, where multiple vasodilatation and vasoconstrictive responses occur [13]. Several studies on animal models and humans indicate that the incidence of repetitive SD correlates with neuronal damage, thus worsening the clinical outcomes. Thus, it is widely accepted that SD constitutes another major contributor to secondary damage [79,80]. Although SD can be modulated by different drugs in intact tissue, for example, by NMDA receptor antagonists, SD waves are generally pharmacoresistant in metabolically compromised tissue, a fact likely ascribed to the lack of deep knowledge of the mechanical aspects of the initiation and propagation of SD in the ischemic brain. Although NMDA antagonists, such as ketamine or MK-801, might attenuate SD in animals after brain injury [81], only few clinical cases have reported that antagonizing NMDA receptors might prevent SD in patients [82]. The fact that targeting excitotoxicity through NMDA receptors antagonists does not result in clear benefits in stroke patients brings even more controversy as to whether NMDA receptors are adequate targets for abolishing SD and attenuating secondary damage. Conversely, SD is considered a strong biomarker for monitoring the clinical evolution of stroke patients [79], but the identification of promising compounds to target SD waves, and their vasoconstrictive effects over the evolution of damage in an ischemic brain, remains a very attractive area for therapeutics.

## 3. Biomaterials and Routes of Administration

Numerous materials have been used in medical applications over the centuries. With the emergence of modern biomedical engineering and the ability to design and produce synthetic polymers or prepare natural materials through standardized methods, we have significantly advanced in the prevention, treatment, and diagnosis of a variety of human pathologies [86]. In the last few decades, there has been an indefatigable search to cover the most pressing needs in biomedicine and tissue engineering. This search includes exploring and characterizing the most highly compatible biomaterials and formats with low cost, easy production, the ability to be sterilized, and reproducible manufacturing. The search for biomaterials able to deliver molecules and factors, interact with cells and tissues, or simply serve as support for skeletal tissues and different organs has been done on a large scale. For example, this search has been fruitful in the fabrication of stents to recanalize clogged vessels, synthetic valves for aortic/mitral stenosis/regurgitation, bone and orthopedic prostheses, intraocular lenses, immunobiology, cell and drug delivery systems, or general materials for in vitro diagnosis [87,88,89]. Biomaterial-based biomedical applications have grown exponentially in parallel with the development of better artificial/natural polymer designs, with a progressively greater capacity to innocuously adapt to tissues and organs. In the case of the central nervous system (CNS), the applications of biomaterials are very versatile. For example, popular polymers like hydroxyapatite or poly-methylmethacrylate have been used for cranioplasty and skull reconstruction after craniectomy in patients that have suffered ischemic and hemorrhagic stroke [90,91]. Endovascular embolization with platinum coils coupled with hydrogels has been performed as an alternative to neurosurgical clipping for the treatment of intracranial aneurysms [92]. Other polymers, such as polyvinylpyrrolidone, have been used to coat endovascular catheters and vascular devices [93].

Different biomaterials have been used as adjuvants for drug and cell delivery to cover the lack of clinical efficacy of many neuroprotective agents based on negligible systems of drug delivery and to achieve therapeutic doses during the intervention window. The majority of classical neuroprotective approaches ignore the optimal time point for drug application, as well as the length of duration and quantity needed for a particular neuroprotective agent to remain active to achieve its maximal clinical response. After injury, over time, positive and negative signalling emanates from the brain [94,95]. Due to this fact, particular compounds might have antagonistic effects on the time of application causing detrimental or neuroprotective actions. For example, early inhibition of the chemoattractant C-X-C motif chemokine 12 (Cxcl12) might prevent the infiltration of peripheral leukocytes into the brain, diminishing inflammation and leading to functional recovery [96]. However, the inhibition of Cxcl12 at later time points after stroke would prevent tissue remodeling because this cytokine stimulates the migration of endothelial and neural progenitors towards peri-lesional and lesional regions. Although it is assumed that the modulation of activity in molecules related to inflammation/oxidative stress/excitotoxicity pathways produces neuroprotection during the window of intervention, the efficacy of such neuroprotective treatments is limited by a profound decay of activity linked with the poor stability and rapid degradation of majority of neuroprotective compounds. These factors, together with the restricted permeability of the BBB, the amount of unbound drug compared to the drug bound to non-target intra and extracellular molecules, and the limited drug distribution into the brain, indicate that the drug concentration generally falls below therapeutic levels, thus narrowing the time of therapeutic action [97].

### 3.1. Intracranial Administration

As stated above, the BBB represents one of the most important restrictions to the passage of molecules towards brain parenchyma when they are administrated systemically [98]. It has been estimated that nearly 95% of small molecules do not cross the BBB [98]. Two main mechanisms might help molecules pass through the BBB: free diffusion by crossing the lipid-composed membranes of endothelial capillaries and transportation through specific receptors. The latter process represents the main mechanism for the transport of small polar molecules, such as amino acids, glucose, or certain anions and cations towards the brain [99]. These specific mechanisms strongly restrict the passage of the majority of molecules. It has been reported that only small (<400 Da) lipid soluble molecules have the ability to cross the BBB. The molecular weight, lipophilicity, and hydrogen-bonding potential strongly influence this process. For example the number of hydrogen bonds is negatively correlated with drug permeation [100]. To circumvent the BBB, different biomaterials have been uploaded with bioactive molecules and directly implanted into the brain. With the intracerebral approach, the location of the implant in cortical or subcortical structures can be reasonably chosen, thus establishing a core of delivery either in the damaged area or in the surrounding tissue to reach therapeutic concentrations. This approach reduces the dose that is needed to produce positive effects and decrease the toxicity associated with systemic approaches that usually require higher doses of drugs. In addition, cerebral implantation increases the drug half-life by preventing the drug’s exposure to the plasmatic proteins and molecules responsible for drug removal. This approach might be particularly relevant for treating focal ischemias. Although different biomaterials and formats can be implanted in the brain, thereby extending drug activities, their biocompatibility and integration with host tissue still constitutes an important concern that needs to be resolved. The cerebral microenvironment is extremely sensitive to minor damage. Even the simple implantation of an inert foreign body, such as the tip of a needle, produces a rapid inflammatory response [101]. Another major disadvantage of cerebral implantation is the invasiveness of surgical procedures required to implant the biomaterial, which can displace healthy tissue, thereby compromising the structure and function of the non-damaged brain regions, especially if the mechanical properties of the implanted materials do not match the specific properties of the brain tissue, which also vary between mammalian species and different brain structures [102,103,104]. To minimize damage, epicortical implants have been performed to release drugs directly onto the brain’s surface, avoiding penetration in deep brain structures [105]. This epicortical strategy is interesting when taking into account that many stroke patients are treated with decompressive craniotomies and durotomies to relieve intracranial pressure, thus exposing the brain surface [106]. However, these neurosurgical procedures are not usually apply to aged patients as these interventions increase the risk of morbidity and mortality [107]. Both intracerebral and epicortical implantation might establish a gradient of drug concentration from the site of implantation towards other brain areas, limiting the ability of compounds to reach therapeutic doses in the outermost regions (i.e., the subcortical areas). In this context, it has been estimated that, for small molecules, drug concentration diminishes logarithmically for every mm of distance from the capillaries towards the brain parenchyma [98].

### 3.2. Intravenous and Intraarterial Administration

Less invasive procedures have also been explored (for example, intra-venous and intra-arterial routes). Different types of nanoparticles (NPs) have been delivered through these specific routes. Although it has been suggested that a systemic route may extend the drug’s half-life, the restricted permeability of the BBB, which limits the accessibility of the administrated compounds and nanoparticles to the brain, remains a significant concern. Although the BBB is strongly hermetic under physiological conditions, a breach in the BBB occurs in minutes after a brain injury and lasts for days. After a stroke or traumatic brain injury the temporal course of impaired BBB permeability is complex, the permeability of the BBB is substantially increased in a biphasic way, with maximal permeability a few hours after stroke, followed by a decline and subsequent increase of permeability between three and seven days after the insult [108,109]. In mice, transient ischemia causes substantial BBB opening, mainly at 6 and 72 h, similarly following biphasic evolution [110], a result previously found in other mammalian species [111]. Although some patients may show impaired BBB permeability for weeks, for the majority of stroke patients, the permeability of the BBB quickly returns to its physiological values. This creates a temporal window of opportunity for bioactive compounds that usually do not cross the BBB to reach the brain through the use of different nanomaterials coated with therapeutic compounds that are systemically administrated. A widely used coadjutant for drug delivery is the NP format, which may increase the stability of drugs in circulation by creating a shell against rapid degradation and controlling the progressive drug release. In an interesting study, the systemic administration of PEGylated polystyrene NPs of a wide size (40–1000 nm) produced the maximum accumulation of NPs in the brain one hour after injury [112]. However, at later time points (13 h), only the smallest particles were clearly detected in the brain. This study suggests that, although the permeability of the BBB is impaired after injury, the BBB still has some limitations in allowing particles and biomolecules to enter the brain with absolute freedom. In addition, therapeutic intervention at very early time points after BBB opening (for example, 1 h post-injury), precisely when maximal NP accumulation is achieved in the brain [112], is not practical for a late clinical diagnosis. Because less than 5%–10% of systemically administered NPs might reach the brain as the target organ [113], and because most NPs are eliminated by glomerular filtration at the renal level, the non-invasive aspect of intra-venous/arterial administration is counteracted by the limited ability of the NPs to reach the brain, even with a breach in the BBB.

### 3.3. Intranasal Delivery

A third level of administration is the intranasal pathway. The anatomy of the nasal cavity might allow the passage of substances directly towards the brain through this particular route [114]. Neuroprotective agents could theoretically pass from the nasal cribriform plate and neuroepithelium to the brain via the olfactory and trigeminal nerves, thus bypassing the BBB [115]. However, the olfactory epithelium has, in general, reduced permeability to large molecules or polar molecules under non-injured conditions, although hydrophobic small molecules might pass the nasal epithelium with fewer restrictions. Although this route is less invasive than intraparenchymal administration, the rate of diffusion is still highly reduced, thereby limiting the amount of total drug entering the brain to reach therapeutic doses in the area of interest. It is estimated that only 1% to 10% of administrated drugs, depending on their chemicals’ structure and size, can target the brain through this route [116]. Due to this limitation, several strategies have been developed to improve the transport of different substances through the intranasal route, such as the use of NPs decorated with ligands with an affinity for olfactory epithelium receptors [117]. However, anatomic differences in the olfactory epithelium between humans and rodents might account for the differences in the permeation rates between species. How this route works in humans, however, is largely unknown [21].

**Figure 2:**
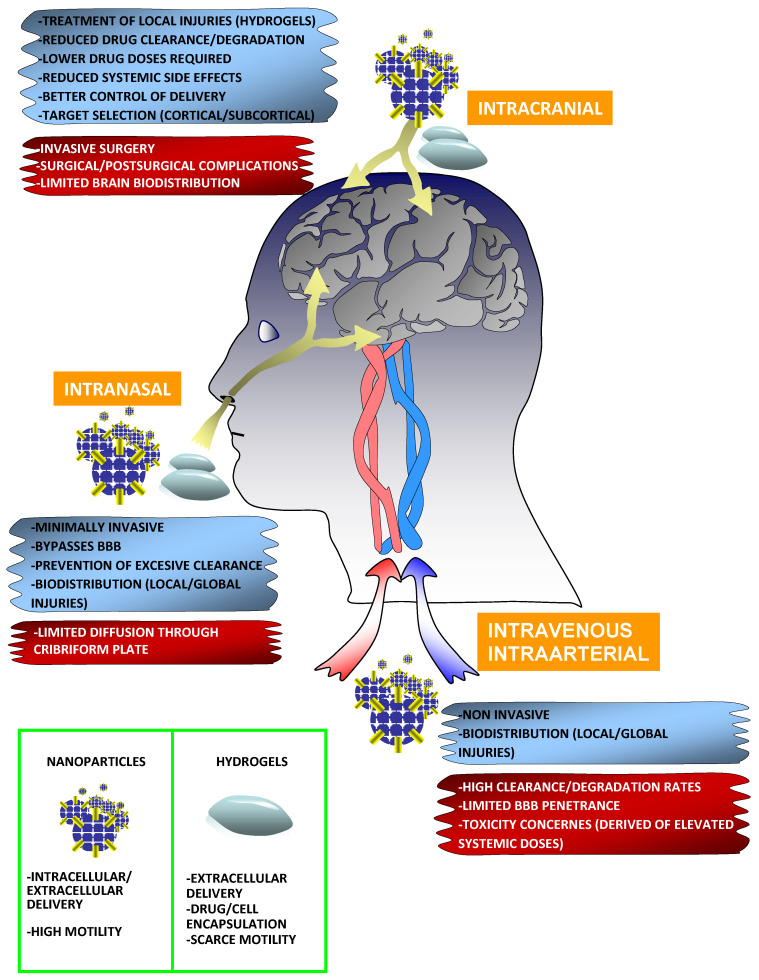
Main routes to target the stroke brain with biomaterial-based nanoparticles and hydrogels. Intracranial (stroke cavity, epicocortical, and intracerebral), intranasal, and intravenous/intraarterial routes are the commonly used to target the brain with distinct biomaterials and formats. The advantages and limitations of each specific route are framed under a cyan and red background respectively. Nanoparticles (NPs), such as micelles, liposomes, dendrimers, and inorganic NPs are strongly versatile and can be administrated through all known routes to treat both focal and global injuries. NPs are more dynamic, with better dispersion and biodistribution properties than hydrogels, which usually hold static positions in the area of implantation. NPs might also act on extracellular and intracellular targets. In addition to the delivery of drugs/factors, different stem cells and differentiated cells with neuroprotective abilities can be encapsulated in the interior of hydrogels. The limited diffusion, reduced BBB penetrance, and excessive clearance of NPs might be overcome with biofunctionalization strategies, for example, incorporating camouflage molecules (PEG or cellular shell membrane fragments), or decorating the NPs with particular ligands to target receptors in the brain endothelium or olfactory epithelium.

To overcome the rapid dilution and degradation of neuroprotective drugs, most biomaterial-based strategies use NPs and hydrogels, which, in general, have demonstrated significant neuroprotective preclinical benefits with respect to the administration of free molecules. In the context of stroke, hydrogels have been preferentially developed to deliver drugs/factors directly into the brain, especially to treat focal injuries, with precise deposition of this biomaterial in the areas of interest (i.e., in the stroke cavity or in peri-lesional areas). However, NPs are more versatile because they can be administrated through all imaginable routes, including systemic (intravenous, intraperitoneal, and intranasal) and cerebral routes, to treat focal and extensive (global) injuries, with greater biodistribution but higher clearance rates (Figure 2). In addition, NPs might also target intracellular components by crossing the cellular membrane. In the next two subsections, we will discuss the relevant properties of NPs and hydrogels in relation to their potential uses for targeting secondary damage after brain injury.

### 3.4. Nanoparticles

A very attractive material format for biomedical applications is the NP, which usually results from the association of bioactive compounds and molecules, drugs, peptides, protein factors, antibodies, deoxyribonucleic acid (DNA), ribonucleic acid (RNA), and interfering RNA, with a core structure formed by natural or artificial polymers, lipids, or a combination thereof. For targeting stroke, most studies to validate the neuroprotective efficacy of drugs/factors delivered from NPs have been carried out in rodent models and very rarely in non-human primates (Section 4). To the best of our knowledge, no NP-based applications have been used to target the human stroke brain. However, in non-stroke clinical trials different NPs based on micelles, liposomes and various polymer composites have been already used to deliver therapeutic compounds [118]. Apart from drug/factor delivery, NPs have also been used as contrast agents for brain imaging and diagnosis in rodents, and less frequently in human and non-human primates [119,120,121,122,123,124]. Some studies have focused on developing multifunctional NPs (for example, for drug delivery and imaging) [125,126].

NPs work on a nanometric scale (~10–500 nm) and are thus able to interact with tissues extra- and intra-cellularly. However, such interactions need to be strictly controlled since non-specific interactions and retention in several tissues reduce the number of free NPs available for binding to the selected targets. NPs show, in general, good stability and can be sterilized by different methods, including γ-irradiation. For sizes of NPs, ranges between 10 and 100 nm are compatible with the diameters of blood vessels in the brain and the sieving coefficient for the glomerular capillary wall in the kidney for subsequent NP clearance, although large NPs might have limited diffusion rates in the brain parenchyma. NPs can be constructed with different biodegradable and compatible materials and polymers, including chitosan, poly-lactic acid (PLA), poly-lactide-co-glycolide (PLGA), poly-methyl methacrylate, poly-N-isopropylacrylamide (PNIPAM), poly-butyl cyanoacrylate (PBCA), poly-isohexyl cyanoacrylate (PIHCA), gelatine or albumin, and others. These materials influence the efficacy of drug encapsulation and subsequent in vivo delivery in different ways. The sizes, charges, and shapes of NPs can be tuned to modify the rate of cellular uptake, transport, biodistribution, and kinetics for the fast or slow release of different compounds to reach extra-or intra-cellular compartments, depending on the specific target location. NPs have been conventionally classified as different types, including (1) polymeric NPs (i.e., dendrimers, micelles, chitosan, PLGA, and PGA), (2) lipid-based NPs composed of fatty acids and triglycerides (i.e., liposomes), (3) inorganic NPs formed by silicon, pure metals, and alloys, or (4) hybrid NPs.

#### 3.4.1. Functionalization of Nanoparticles

The actual technology with NPs allows their surfaces to be decorated with chemical and biological motifs to prevent the rapid decay of the drug concentration in circulation as a consequence of excessive degradation and poor stability due to the body’s clearance/excretion and metabolism. NPs can be externally decorated with targeting ligands, and it is possible to change the affinity and density of these molecules (e.g., antibodies) to identify specific subsets of native or pathological cells in tissues and organs and ensure the subsequent delivery of NPs in these selected targets. The particle size, chemical structure, and zeta potential can be engineered to create a wide spectrum of particles with different properties. As noted above, brain injury compromises the BBB, making it more permeable to compounds that usually would not pass the BBB under physiological conditions. This increase in BBB permeability gives NPs a temporary opportunity to gain access to the brain through a Trojan-horse strategy when NPs are less-invasively and systemically administrated, although the NP size might influence the rate of internalization and diffusion into the brain, as well as the amount of drug released. Although brain injury produces a breach in the BBB, this does not necessarily mean that the brain’s endothelium permeates almost every specific compound or NP type. The surfaces of NPs might be modified covalently by incorporating certain molecules, such as Poly-ethylene glycol (PEG) and surfactants, or by adsorbing targeting molecules; the latter facilitates NP uptake into the brain through receptor-mediated endocytosis mechanisms. For example, NPs might be decorated with apolipoprotein E (ApoE) fragments or anti-transferrin antibodies to cross the BBB though interactions with low-density lipoprotein (LDL) and transferring receptors, respectively [127,128]. Uptake is also favored when NPs are decorated with particular ligands to target glutathione or glucose transporters present in the endothelial barrier [129]. Functionalization with surfactants, such as polysorbate 80, pluronicP85, or poloxamer 188, drastically enhances the ability of NPs to cross the BBB via active transcytosis mechanisms [130]. These surfactants present in the NPs’ surface adsorb the Apo A-I and/or Apo E present in the plasma to facilitate their subsequent interactions with LDL receptors. In one study, for example, polysorbate 80 coated poly(butylcyanoacrylate) NPs enhanced the brain concentration of low-molecular weight neuroprotective molecules, such as tacrine or rivastigmine [131,132]. Transcytosis is an active mechanism of NP delivery that is very specific and can also enhance the intracellular delivery of bioactive compounds. In contrast, the passive entrance of NPs into the brain via leaky BBB vasculature as a consequence of injury results in non-specific targeting, but a large number of NPs can pass into the brain. NPs have also been employed to deliver high-molecular factors and enzymes. In one study, albumin-fluorescein isothiocyanate (FITC) delivered from PLGA NPs modified with a particular heptapeptide sequence (g7) was found in the brain of wild type mice as early as 2 h after intravenous injection, while the amount of albumin-FITC and NPs in the brain was even greater in mice with mucopolysaccharidosis and impaired BBB permeability [133]. In another study, high-molecular weight neuroprotective factors, such as brain derived neurotrophic factor (BDNF) released by PEG-poly-L-glutamate-(PGA) diblock copolymer NPs administrated intravenously, reduced the infarct size and promoted the recovery of neurological dysfunction after transient ischemia in comparison with the application of free BDNF [134]. Nerve growth factor (NGF) adsorbed onto PBCA NPs decorated with polysorbate 80 led to an improvement of deficits in a mouse model of Parkinson’s disease [135]. Curiously, the degeneration of motor neurons has been associated with a decrease in the expression of different transcytosis receptors, for example, transferrin receptor 1 [136]. Thus, particular pathologies might modify the ability of NPs to cross the BBB via receptor-mediated transcytosis. As a very frequently used molecule to decorate NPs, PEG might make NPs practically invisible to the immune system [137]. This is important because NPs rapidly adsorb serum proteins, including opsinins (antibodies, complement factors, and Pentraxins), which mediate opsonisation, thereby enhancing phagocytosis by peripheral macrophages augmenting the clearance of NPs from circulation. Thus, decorating the surface of NPs with surfactants or PEG might prolong the NPs’ time in circulation [138]. PEG-based NPs have been, for example, employed clinically as anti-tumoral therapies. If NPs survive the hostility of the blood serum and cross the restricted BBB, they can be modified with certain ligands to target specific subsets or neural cells into the brain parenchyma. This is the case for the amino acid sequence “CLEVSRKNC”, identified via the screening of a phage library, which might facilitate the mobilization of NPs towards ischemic penumbra areas due to the special affinity of this peptide sequence to binding neuronal cells at risk of being damaged [139,140].

The stability and clearance of the NPs in circulation might be influenced by the NP charge. The surfaces of cells, including the endothelium lining the blood vessels, contain negative charges that can repel NPs with negative potentials. However, NPs with excessive amounts of both positive and negative charges might also increase the phagocytic activity of monocytes/macrophages, thereby diminishing the quantity of NPs. The positive charging of NPs with cationic peptides and different molecules allows the interaction with the BBB surface, which is negatively charged. However, highly charged anionic and cationic NPs might also disrupt the BBB [141]. Positive potentials also promote NP aggregation with the negatively charged proteins present in serum, thereby increasing the risk of embolisms. Thus, a neutral charge is desirable to provide better stability by diminishing excessive clearance and toxicity at the expense of a minor rate of infiltration towards the brain [142]. The coating of NPs with PEG might bring the zeta potential closer to zero values [112]. The zeta potential also influences the rate of intracellular internalization. Because the plasma membrane is negatively charged, positively charged particles can enter more easily than negative particles. Another concern is related to tolerability. In most preclinical research, the functionalization of NPs and the core itself are not associated with substantial toxicity concerns, although there still exists a potential risk that particular materials and biofunctionalization can produce toxic effects [143,144]. In humans, the possible adverse effects of particular NPs and biomaterials at the systemic and cerebral levels are in general largely unknown.

#### 3.4.2. Dendrimers

Very specific carriers for drug delivery are dendrimers. This type of NP has a tree-like branched topology formed by a core, from which multiple layers of branches emerge and incorporate distinct functional groups that can bind different neuroprotective molecules and factors. This particular structure and the versatility of these NPs’ functional groups allow the encapsulation and covalent binding of a great variety of hydrophilic and hydrophobic molecules. A popular dendrimer is poly-amide-amine (PAMAM), which, due its commercial availability, has been used in multiple applications, including the treatment of stroke. This dendrimer is made of repetitive subunits of amide and amine groups and shows greater biocompatibility than other dendrimer isoforms [145]. However, toxicity concerns have also been reported in specific PANAM variants carrying cationic groups. Although the biological tolerability and lifetime in circulation increased after decorating these dendrimers with PEG, PEG did not completely suppress toxicity, especially when higher proportions of PANAM are were used [145].

#### 3.4.3. Liposomes

Liposomes are spherical NPs that are formed by an amphiphilic lipid bilayer. Liposomes can encapsulate both hydrophilic and hydrophobic molecules, including neuroprotective drugs, DNA/RNA, peptides, and recombinant or natural proteins. Changing the lipid composition, size, and zero potential might increase the lifetime in circulation and within the brain. Similarly, PEG or the incorporation of gangliosides might prolong the time in circulation and reduce blood clearance. It is also possible to reconstitute liposomes by incorporating recombinant ligands for specific receptors to mediate brain uptake through endothelium receptor transcytosis. In one study for example, Dimyristoylphosphatidylcholine-based liposomes were decorated with APOE to carry α-mangostin, an inhibitor of Amyloid-β (Aβ) oligomer formation. These NPs, with size ranges of ~30/50 nm and zeta potentials of −10/−20 mV, were able to cross the BBB after intravenous administration, reaching the cortex and hippocampus [146]. In APPswe/PS1ΔE9 Alzheimer mice, as a model of neurodegeneration, these NPs decreased amyloid deposition and microgliosis, leading to improvement in spatial learning and memory capacities [146]. Alternative approaches to increase the entrance and delivery of liposomes in the brain have also been reported, for example, via the previous osmotic disruption of the BBB using mannitol [147] or by surface modification with several ligands to promote receptor-mediated endocytosis, such as ApoE or molecules that target BBB transferrin receptors [148,149]. As the main handicaps, it has been established that liposomes have a poor control of time release, as well as limited intracellular delivery, although these concerns are overcome by the versatility of NP applications for drug delivery in stroke and several neurodegenerative disorders.

#### 3.4.4. Micelles

Another type of NP around 5–50 nm in size are micelles, which are composed via self-assembly in water from amphiphilic molecules, with hydrophilic groups facing the outside and hydrophobic ones inside the micelle forming the core. Self-assembly in water occurs at a defined concentration of surfactants to achieve minimal surface tension and the formation of micelles (critical micelle concentration). Micelles are readily able to encapsulate and deliver poorly soluble molecules. Their hydrophilic features also allow better integration with aqueous extracellular media, and micelles are relatively easy to produce. In the context of neurological disorders, micelles have been used, for example, to release anti-epileptic compounds, with a limited capacity to cross the BBB. For example, a copolymer of pluronic acid derivates was used to deliver clonazepam, an anti-convulsant drug that potentiates GABAergic signalling, thus reducing excitability [150]. With the most optimal micelle formulation, a sustained release of clonazepam was obtained over an interval of eight hours, although this contrasted with the peak of maximal concentration of this drug in the brain; approximately 30 min after intranasal administration [150]. Interestingly, very few concentrations of clonazepam were detected in the brain after the intravenous administration of micelle–clonazepam or the delivery of the free drug, thus highlighting the efficacy of the intranasal route for this specific approach. In mice, this strategy offered better protection against epilepsy in a model of pentylenetetrazole-induced epilepsy [150]. In other example, Pluronic-derived micelles reconstituted with phosphatidylcholine and polysorbate80 have also been used to deliver Nimodipine, a calcium antagonist that reduces cerebral vasospasm in subarachnoid haemorrhages [151]. More recently, copolymers of PEG and PLA were used to fabricate self-assembling micelles in a nanometric range to release the anti-oxidant and ROS scavenger compound Edaravone [152].

### 3.5. Hydrogels

Hydrogels offer an alternative perspective on pharmacological delivery relative to NPs, although various strategies have simultaneously employed a combination of NPs and hydrogels, for example, by encapsulating NPs carrying neuroprotective agents in the interior of hydrogels [153,154]. Most hydrogel-based applications seek to implant the hydrogel in a defined cerebral location while different therapeutic factors are gradually delivered from this particular implant area, thus establishing a gradient of concentration from the site of implantation towards the injured tissue. The therapeutic opportunity of hydrogels to neuroprotect the stroke brain is inferred from the positive outcomes observed in animal models (Section 4). At present, there are no known applications to target the human stroke brain with hydrogels, although some initial approaches have been performed in non-human primates, exploring the tolerability of several hydrogels formulations (PEG, hyaluronic acid) implanted in the ischemic and non-ischemic brain [155,156]. Fortunately, the rapid evolution and characterization of hydrogel-based materials for drug delivering in other mammalian species (mainly rodents) reinforces our optimism to develop advanced hydrogels for clinical translation.

Hydrogels are formed by immersing a polymer or a mix of polymers into an aqueous solution, thereby producing an insoluble three-dimensional gel state. Hydrogels have a strong affinity for adsorbing water molecules, and most of their composition is water (>90%). This special architecture is compatible with soft tissues/organs, such as the brain; given that the mechanical properties of hydrogels, usually modifiable depending on polymer concentration and crosslinking-density, generally match the compressive modulus of brain, estimated in the range of 25–50 kPa in rodents and 2–10 kPa in humans [103,104,157]. For brain stroke, hydrogels have been preferentially designed for direct cerebral administration, although hydrogels have also been applied via the intranasal route [158]. In cerebral applications, hydrogels can be injected in a pregel state (liquid) to achieve in situ gelation over a time window of a few minutes [101]. This strategy reduces invasiveness, prevents subsequent damage of viable functional tissue, and is very appropriate for cell encapsulation. Different hydrogel-based biomaterials have been implanted in the striatum [84,159], in the stroke cavity [160,161], or epicortically above the brain’s surface [162,163]. A priori, the implantation of static hydrogels could be more able to treat focal injuries, although alternative approaches should be explored for global damage caused by severe stroke or neurodegenerative disorders, affecting several brain structures, such as the cortex, hippocampus, and striatum (e.g., Alzheimer’s disease).

When comparing both biomaterial formats, NPs are more versatile and can be administrated through all known routes (intraperitoneal, intravenous, intranasal, or cerebral). NPs show better diffusivity properties than hydrogels, allowing them to reach almost every brain region, although this dynamism might be counteracted by the sizes of NPs, the medium viscosity, and their non-specific interactions with the extracellular components of brain parenchyma [164]. For example, NPs larger than 200 nm show limited diffusivity through the cerebral area. It has been reported that PEG-coated NPs with sizes near 100 nm and zeta potentials close to zero show efficient rates of diffusion through brain tissue [165]. In agreement with this study, NPs smaller than 100 nm were efficiently able to target and distribute through the injured brain after intravenous administration [166]. However, even in smaller NPs, the diffusion rate and distribution across the brain area can be affected by NPs’ composition. In addition, NPs are subjected to higher clearance rates than hydrogels. Similar to NPs, hydrogels can be designed and fabricated with the incorporation of natural or artificial polymers, or a combination of them. Thus, polymers formed by chitosan, PEG, PLGA, hyaluronan, collagen, methylcellulose, alginate, and Matrigel are very popular [167]. These hydrogel-based biomaterials have been used for the delivery of different neuroprotective and neurotrophic agents, for example neurotrophin-3, ephrin-A, BDNF, and erythropoietin, among others [154,168,169,170,171,172].

#### Mechanical Properties, Degradation and Dynamic Hydrogels

Specific hydrogel formulations can define their mechanical and biological properties and their capacity to integrate with the host tissue. The particular properties of hydrogels might make them ideal not only for drug delivery but also for the encapsulation of terminal and undifferentiated cells. In general terms, hydrogels provide suitable environments for the exchange of oxygen, nutrients, and waste products between the encapsulated grafted cells and the recipient tissue. Hydrogels can also facilitate the delivery of different bioactive molecules and factors released directly from the encapsulated cells (secretome), such as factors with repercussions as modulators of inflammation, neurogenesis or angiogenesis [173]. Far from being a passive structure for the delivery of biological factors, due to its properties, a hydrogel can allow the encapsulated cells to continue interacting with the host tissue to restore the functional circuits of damaged tissues and organs [174]. Thus, in the context of cellular therapies, it is possible to tune the polymer concentration and cross-linking density to select the mechanical properties of a hydrogel and its level of graft integration with the recipient [175]. For example, it is feasible to create highly compartmentalized hydrogels with very strict barriers to prevent donor cell–host cell contact, thus avoiding the entrance of inflammatory and harmful signals, which are usually present in the damaged tissues, towards the encapsulated cells [84]. Alternatively, it is possible to design more open systems to achieve large-scale integration between donor and host components [176].

In general terms, synthetic materials can efficiently reproduce the mechanical and physical environment of the brain tissue relative to hydrogels constructed with natural materials. To better mimic biological environments, hydrogels can even be formulated with adhesion-mediating molecules, such as the tripeptide Arg-Gly-Asp (RGD), the principal adhesive ligand in fibronectin and other extracellular matrix proteins, such as collagen, laminin, and vitronectin. Hydrogels with higher polymer content and cross-linking density usually produce stiffer materials that generally are more resistant to biological degradation. The vulnerability of a polymer to degradation depends on its structure and specific hydrogel formulation, for example, due to the presence of hydrolyzable ester and amide groups. For particular applications, it might be interesting to design hydrogels with controllable degradation, for example, incorporating motifs sensitive to proteases and metalloproteinases, whose levels of activity increase under pathological conditions. Degradation can also be induced by other advanced approaches, for example, through photolytically degradable hydrogels [177]. In this study, photodegradation was used to temporally control the crosslinking density and transform highly compartmentalized hydrogels in more open platforms. This type of dynamic conversion might be applied at specific time points after injury, depending on the pathological response of the host environment [177]. Additional reports illustrate the progressive evolution of hydrogels towards more dynamic structures [178], for example delivering immobilized molecules using photonic stimulation [179] or in response to increasing levels of MMP-9 in the brain [180]. Interestingly, the cross-linking density, type of polymer, and content of free and bound water not only influence the mesh size but also the permselectivity properties of every hydrogel to sustain drug delivery over hours or days, depending on each application, the type of injury, and the time of intervention.

### 3.6. Therapeutic Potencial of Biomaterials

In addition to its potential for drug release and cell therapy, several studies have shown the special ability of different hydrogel and NPs formulations to mitigate, by themselves, the inflammation and oxidative stress caused by brain injury. For example, when hydrogels of the extracellular matrix from a porcine urinary bladder were implanted in the stroke cavity, they promoted the infiltration of different inflammatory and neural cell populations and neovascularization [181,182]. At concentrations above 8 mg/mL, these hydrogels produced a significant polarization of microglia towards anti-inflammatory phenotypes, which are involved in brain tissue remodeling and repair after injury. Hyaluronic acid-based hydrogels implanted in the stroke cavity have been linked to reduced inflammation, creating a permissive environment for neural progenitors that migrated from neurogenic niches towards the injured site [161]. These neuroprotective effects have also been observed with NPs. For example, when hydrophilic carbon NPs decorated with PEG were intravenously administrated, they showed anti-oxidant properties, thereby reducing the infarct volume and leading to functional recovery in a model of transient ischemia in rats [183]. In another example, selenium NPs decorated with PEG and OX26 antibodies to target the transferring receptor were able to reach the injured brain after intraperitoneal injection [184]. These selenium NPs (zero potential +1.17 mV) reduced the brain edema and decreased the infarct size in a model of transient ischemia in rats. These NPs did not cause serious toxicity concerns in several organs that were histopathologically analyzed, such as the lung, spleen, liver, and kidney. The neuroprotective effects of these selenium NPs were ascribed to their specific targeting of several pathways involved in oxidative-stress, inflammation and apoptosis [184]. It has been suggested that the neuroprotective and neurogenic effects induced by particular hydrogels and NPs devoid of neuroprotective/neurogenic agents might be supported by their mechanical, structural, and chemical characteristics, although the molecular/cellular mechanisms have not been completely elucidated.

## 4. Neuroprotective Biomaterials for Brain Injury

In general terms, biomaterials can sustain the progressive release of therapeutic drugs and factors preventing the early degradation and clearance of vulnerable compounds. This general characteristic might prevent fluctuations in drug concentrations, ideally reaching zero order kinetics, thus avoiding dangerous increases or decreases in drug concentrations to achieve concentrations that are relatively constant, within therapeutic ranges. While the highly compartmentalized nanostructures of many biomaterials and formats might have consequences on the fitness of the cells seeded within it, this excessive compartmentalization does not generally limit the delivery of encapsulated drugs and factors, although this delivery might be affected by the non-covalent interactions of distinct encapsulated biomolecules with the constituent polymers and materials used to fabricate each particular NP and hydrogel. For stroke, most biomaterial-based therapeutic applications have been focused on the use of neuroprotective, angiogenic, and neurogenic agents. However, some nanoengineering strategies have been acutely applied very early after stroke to re-establish the impaired blood flow caused by the obstructive process. For example, in an interesting study, PLGA NPs were able to respond before an increase in blood pressure (shear stress) was produced, thus disintegrating and releasing the anti-thrombolytic agent t-PA, which, as mentioned previously, remains the gold standard compound for treating ischemic stroke or myocardial infarction [185].

### 4.1. Hydrogels and Nanoparticles to Target Angiogenic and Neurogenic Niches

The so-called neurovascular unit is a frequently used term to illustrate the complexity of nervous tissue, formed by a subset of undifferentiated and terminally different cells, which include stem cells and neural precursors, neurons, astrocytes, oligodendrocytes, microglia, pericytes, and vascular cells anchored to extracellular matrix components that provide physical support and mechanical stability, as well as metabolic and electrical cooperation [186]. Although the classic perspective notes that pericytes and vascular cells are key components of the BBB, the remaining neural cells also interact with the brain’s microvasculature, thus helping to regulate the function and strict permselectivity of the BBB [187]. Without vascular cells and neovascularization, it is not possible reconstruct the damaged brain tissue through real tissue replacement and/or tissue remodeling to restore loss of function, thus guarantying the long-term survival of new/remodeled tissue [188]. Many stimulators of angiogenesis are not only essential to enhance neural cell progenitors’ migration towards the ischemial penumbra, but also permit the reestablishment of BBB permeability, with additional neuroprotective effects [188,189]. Different advanced biomaterials have been preclinically assayed with this intention. For example, alginate/collagen hydrogels formulated as microspheres have been used as micro-factories for the release of fibroblast growth factor -2 (FGF-2). This biomaterial induced the progressive and sustained release of this angiogenic factor for at least one week, enhancing vascularization in zebrafish embryos [190]. Neovascularization has also been achieved after the brain implantation of the vascular endothelial growth factor (VEGF) released by PLGA microparticles [191]. In stroke rats, this biomaterial-VEGF, in combination with neural stem cells, facilitated the survival, attraction, and migration of endothelial cells from peri-lesional tissue towards the biomaterial implanted into the stroke cavity, forming tube-like vascular structures [191]. Post-stroke angiogenesis and tissue regeneration have been induced with other sophisticated polymers carrying several therapeutic factors. For example, Hyaluronic acid (HA)-based hydrogels loaded with PLGA-VEGF and PLGA-angiopoietin-1 microparticles [192]. In vitro, these advanced polymers sustained the release of both factors for at least 12 days, although near 75% of the factors previously encapsulated were trapped at this temporal point. In vivo, the implantation of this composite in the stroke cavity of mice with permanent ischemia stimulated long-term angiogenesis, enhancing the recovery of post-stroke sensorimotor deficits [192]. A release system based on a blend of capped (hydrophobic ester end-groups) and uncapped (hydrophilic carboxyl end-groups) PLGA particles sustained the gradual delivery of a RhoA inhibitor (CE transferase) for at least 25 days [193]. This is an interesting strategy based on the specific pro-apoptotic signaling role of RhoA, whose inhibition constitutes an attractive paradigm for the treatment of central nervous system injuries, including stroke. A pioneering study was performed on the delivery of basic fibroblast growth factor (b-FGF) and epidermal growth factor (EGF) from a polymer composite of gelatine and 3-(glycidoxypropyl) trimethoxysilane [194]. After focal brain damage, the release of both factors from this advanced scaffold induced brain tissue remodeling based on a significant increase in neural cell proliferation and migration, which was mostly preferentially restricted to glial cells. Other effects were related to angiogenesis and neural structural changes (dendritic growth), although the kinetic release of both factors from this biomaterial composite was not specifically assayed [194]. These previous examples illustrate how different materials and formats are being used to stimulate the real tissue regeneration of damaged areas (tissue replacement) and peri-lesional remodeling to enhance functional recovery. This type of approach usually requires long periods of continuous stimulation and the sustained delivery of neurogenic/angiogenic factors. However, neuroprotective agents should a priori be delivered in earlier and shorter temporal windows after injury, in a range of hours to a few days (~7 days), during the acute phase of stroke [31].

### 4.2. Hydrogels and Nanoparticles to Target Inflammation

The research related to neuroprotective strategies using different biomaterials is extensive, and most studies have been performed at the preclinical level in ischemic and traumatic brain injury (TBI) rodent models (Table 1). For example, a popular biomaterial commonly employed is chitosan. This polymer has been used for the persistent release of different biomolecules, such as neurotrophin-3 [169]. After brain injury in rats, this molecule-biomaterial combination exerted a strong anti-inflammatory effect based on the inhibition of leukocyte infiltration from peripheral blood to the brain, limiting the activation of microglia [169]. The minor inflammatory and permissive brain microenvironment induced by neurotrophin-3 favored neovascularization and stimulated endogenous neurogenesis while also promoted the migration of neural progenitors from neurogenic niches to lesional and peri-lesional areas, which differentiated into terminally functional neurons. All these mechanisms are essential for creating new circuitry in lesional and peri-lesional tissue. The philosophy of using neurotrophin-3 in combination with chitosan carriers has been extended to other scenarios, for example, after spinal cord injury [195]. Other molecules have been released through distinct biomaterials, exerting significant anti-inflammatory effects and stimulating endogenous neurogenesis. This is the case for Cyclosporin A. This immunosuppressant drug has been delivered through PLGA particles dispersed in a blend of hyaluronan and methylcellulose [153]. This advanced biopolymer was implanted epicortically in the brains of stroke rats, thus reducing the invasiveness of alternative administration routes, such as the intra-cerebral or intra-ventricular routes. In this study, Cyclosporin A was gradually released in the injured brain for at least 14 days. The effects of Cyclosporin A clearly increased the neural progenitor cells’ survival, proliferation, and migration to the peri-lesional tissue, although recovery after injury in response to this treatment was not specifically examined [153]. Reported by the same group, Cyclosporin A delivered epicortically from the same biomaterial composite in a non-damaged mouse brain produced significant amounts of this factor for more than 25 days in brain regions 1 mm below the cortical surface [162]. These studies illustrate the importance of analyzing, for every specific neuroprotective compound, the temporal patterns of delivery, as well as the biodistribution (spatial) in cortical and subcortical structures. This is relevant considering that not all types of stroke are similar in terms of their location (affected regions); moreover, differences in drug biodistribution might affect the efficiency of particular drugs to treat certain forms of injury. The same biomaterial composite based on PLGA particles dispersed on hyaluronan and methylcellulose was used to sequentially release erythropoietin and EGF, two molecules with known neuroprotective and neuroregenerative capacities. When administrated epicortically in mice with focal stroke in the endothelin-1 model, both molecules showed tissue repair potential based on a significant increment in the survival and proliferation of neural precursors from neurogenic niches [154]. This strategy was also associated with minor astrogliosis and microgliosis. In contrast, the anti-inflammatory and neurogenic capacity of erythropoietin and EGF was even smaller when both factors were administrated by intracerebroventricular injection [154]. Similar to Cyclosporin A, a significant advantage of this advanced polymer is that it provides the gradual release of both factors, achieving higher concentrations in an interval of 7–21 days after the biomaterial’s implantation. Similarly, in a neonatal ischemic model, the intraperitoneal injection of erythropoietin delivered from PLGA nanoparticles caused a significant reduction in infarct volume that translated into post-stroke behavioral improvement [196]. A similar neuroprotective effect could only be reached with doses ~16.6 times higher of the free molecule. This latter effect is a general observation of using distinct biomaterials and highlights the efficacy of employing distinct polymers to achieve better post-stroke outcomes with smaller doses of the total administrated drug. The neuroprotective effect of erythropoietin has also been tested in other models of brain injury (hemorrhagic strokes) using formulations based on PLGA [197] and PBCA [198] NPs. Another interesting neuroprotective molecule is Osteopontin, which is strongly upregulated after brain injury. Different studies have reported that Osteopontin plays anti-inflammatory, anti-apoptotic, and neurogenic roles and participate in tissue repair mechanisms. The striatal injection of gelatin microspheres loaded with Osteopontin reduced the infarction size of rats submitted to focal brain ischemia. These effects were considerable even when this treatment was administrated late [199]. The encapsulation of Osteopontin in this polymer provided in vivo sustained delivery of this drug for at least four days after implantation, causing an improvement in post-stroke sensorimotor deficits. Osteopontin has also been intranasally delivered from gelatine NPs, showing significant neuroprotective effects when administrated 6 h after stroke (middle cerebral artery occlusion model in rats), while no effect was detected after the equivalent administration of free Osteopontin [200].

### 4.3. Antioxidant Strategies

PANAM dendrimers have been used to deliver antioxidants and anti-inflammatory molecules, such as N-acetyl cysteine [201]. In in vitro studies, these NPs attenuated the production of free radical nitric oxide (NO) from the microglia in response to lipopolysaccharide (a very common pro-inflammatory molecule). Different evidences have shown the dual neuroprotective and neurotoxic roles of free radical NO, but as commented previously, many studies have reported that the production of NO contributes to exacerbating the post-stroke inflammatory response and the extent of damage, as well as increasing BBB permeability. Thus, for example, inhibitors of iNOS and nNOS have been shown to reduce cerebral edema and infarct sizes in different animal models. In comparison with the application of free N-acetyl cysteine, the sustained release of N-acetyl cysteine through PANAM dendrimers produces significant reductions in NO production, requiring fewer doses of N-acetyl cysteine to achieve the same effects induced by the administration of free anti-oxidant molecules [201]. These types of strategies based on the use of antioxidants released through biocompatible polymers are not only of interest for stroke but also to treat other brain disorders in which inflammation and oxidative stress influence the course of the disease [202]. As commented previously, reactive oxygen species (ROS) are basic mediators of neural inflammation and toxicity, especially in ischemia-reperfusion injury. Thus, oxidative stress and ROS are relevant pathological mechanisms that contribute to extending brain damage after a stroke. Oxidative stress has been combated by approaches based on the delivery of free radical scavengers, such as N-acetyl cysteine (previously described) or superoxide dismutase (SOD). This is because SOD is able to detoxify ROS by converting superoxide anions to H_2_O_2_, while H_2_0_2_ can be converted to water via the action of catalase. Thus, both SOD and catalase have been considered excellent candidates to diminish the oxidative stress caused by ischemic injury. However, the half-lives of SOD and catalase are very short, this limiting their therapeutic uses as free molecules. Two studies from the same group reported the possibility of delivering SOD and catalase in a sustained manner through PLGA NPs to achieve better anti-oxidant stability and prolonged therapeutic effects [203]. In in vitro studies, these PLGA particles sustained concentrations of SOD beyond 90 days. In stroke rats, intracarotid arterial administration of PLGA particles loaded with SOD reduced post-ischemic ROS levels, decreasing the infarct size and improving post-stroke behavioral deficits [203]. The internalization of catalase in PLGA nanoparticles protected human neurons from oxidative stress induced by H_2_O_2_. Significantly, these NPs released large amounts of catalase for at least 30 days, thereby protecting the cell membrane’s integrity and morphology after oxidative shock [204]. In another study, SOD was delivered from nano-polyion complexes, with cationic block copolymers decreasing the infarct size and improving functional recovery after systemic administration, despite few NPs reached the brain in comparison with the rest of organs where they remained trapped, including the spleen, liver, kidneys and lungs [205]. A very original and interesting therapeutic mechanism was also proposed in this study. The authors stated that the efficacy of this treatment was based on the ability of NPs to accumulate in damaged blood vessels within the ischemic hemisphere, thereby contributing to thrombus formation and attenuating the oxidative stress caused by ischemia–reperfusion [205,206]. Another interesting antioxidant compound is Thymoquinone, which plays a role in reducing ROS content after brain injury. However, similar to what occurs with other biomolecules, the strict permeability of BBB limits the entrance of this molecule into the brain’s parenchyma. In addition, Thymoquinone is rapidly eliminated from plasma following intravenous and oral administration, showing relatively slow absorption rates [207]. In an in vitro study, Thymoquinone was gradually delivered from PLGA NPs at an interval of 24 h [208]. In vivo, the intranasal administration of Thymoquinone from PLGA-Chitosan NPs rendered, for at least 72 h, higher levels of this bioactive molecule in the blood and brain compared to when these NPs where injected by the intravenous route. In stroke rats, in comparison with the oral and intranasal delivery of the free molecule, the intranasal delivery of Thymoquinone from PLGA-Chitosan NPs led to a decrease in the infarct volume and better behavioral outcomes. These effects were associated with a significant elevation of the scavenging and anti-oxidant capacity of the brain in response to ROS accumulation after ischemia-reperfusion, since SOD and catalase activity, as well as glutathione levels, were substantially augmented [208]. Chitosan NPs have been used to deliver other neuroprotective compounds, such as venlafaxine and Acetyl-11-keto-β-boswellic acid. Venlafaxine is an antidepressant drug whose intranasal delivery from chitosan NPs significantly increased its uptake to the brain [209]. Acetyl-11-keto-β-boswellic acid is a poorly soluble molecule with a limited half-life in plasma but powerful antioxidant and anti-inflammatory effects. The intravenous administration of this compound incorporated into o-carboxymethyl chitosan NPs produced higher concentrations of this bioactive compound in plasma (at least five times more) compared to the administration of the free molecule [210]. In a cerebral ischemia–reperfusion model, the Acetyl-11-keto-β-boswellic acid reduced neuronal death and decreased the extension of damage, leading to an improvement of behavioral deficits through an increase of the HO-1/Nrf2 anti-oxidative pathways and a decrease in the content of inflammatory molecules, such as NF-kB and 5-LOX [210]. An anti-inflammatory and anti-oxidative stress agent, Fenofibrate, was gradually delivered for at least seven days using PLGA NPs [211]. The intracerebral administration of fenofibrate-PLGA moderately reduced the infarct size in stroke rats, although this effect was not statistically significant, a fact that could not be ascribed to the possible degradation of the drug and was more likely related to the limited biodistribution of fenofibrate in the brain (however, this hypothesis was not tested) [211]. Nitrones are a family of interesting reactive oxygen species (ROS)-trapping compounds that have shown neuroprotective effects in neurodegenerative and cerebrovascular disorders, including stroke [212]. Because nitrones in circulation have a relatively short half-life, their encapsulation in different polymers might increase the durability of therapeutic effects, thus presenting better outcomes. The nitrone NXY-059, a disulfonyl derivate of phenyl butyl nitrone, has shown neuroprotective properties in stroke models. However, this compound failed to exert significant benefits in patients [53]. Whether this lack of efficacy is due to the poor stability of this antioxidant compound in humans versus rodents is unknown. Nitrones are now being preclinically delivered from advanced materials. For example, phenyl butyl nitrones have been stabilized in chitosan NPs decorated with PEG, where significant amounts of this nitrone were delivered over an interval of 24 h. However, the therapeutic efficacy of this formulation was not explored [213]. In contrast, the in vivo neuroprotective ability of the prostacyclin agonist, ONO-1301, has been examined after its subcutaneous delivery through PLGA microspheres [214]. However, drug encapsulation did not produce better benefits compared with the oral administration of the free molecule. In stroke rats, both types of delivery reduced the cerebral edema and extensive damage that lead to similar post-stroke functional recovery after treatment. Considering the clinical context, a remarkable aspect of this study is related to the unique injection of ONO-1031 from PLGA particles in comparison with the repetitive oral administration of the free molecule [214]. Edaravone, a compound approved to treat stroke patients in Japan, has shown neuroprotective benefits by decreasing oxidative stress and inflammation. Due its reduced half-life, the clinical effect of edaravone is likely suboptimal. Biomaterial-based strategies have been developed to overcome this poor stability. In a recent study, edaravone was delivered via micelle NP formulated with an amphiphilic copolymer methoxypoly (ethyleneglycol)-b-poly (D,L-lactic acid) [152]. In addition to edaravone, this specific micelle formulation was decorated with an adenosine 2A receptor agonist to favor its entrance through the BBB and increase its penetration into the cerebral area after intravenous injection. In comparison with the administration of free molecule, the delivery of Edaravone from these NPs reduced the content of ROS and the infiltration of inflammatory cells, thereby reducing the infarct volume and promoting behavioral recovery in a photochemically-induced stroke model in mice [152]. Notably, these positive effects were observed even when the NPs were injected late after stroke, thereby extending the time window of opportunity to rescue the cerebral tissue in risk of damage. This study also assessed a model of permanent ischemia, which mimics a common condition (irreversible occlusion) among stroke patients. Another interesting anti-inflammatory and antioxidant compound, curcumin, has been delivered by different materials to overcome its limited stability in circulation. For example, NPs coated with Polysorbate 80 and lecithins to increase intestinal adsorption were used to deliver curcumin in a model of global ischemia in rats. This therapeutic approach significantly improved the functional recovery of stroke animals based on the analysis of memory consolidation, an effect that was not seen in rats treated with the free molecule [215]. Thermosensitive Pluronic F127/Poloxamer 188-based hydrogels have been developed to deliver curcumin through the intranasal route [158]. In healthy rats, this hydrogel formulation sustained the delivery of this compound for a minimum of 6 h (the time period analyzed), with higher curcumin concentrations in several brain regions compared to the intravenous administration of the free molecule [158]. Neuroprotection has also been achieved with additional technology, e.g., via the incorporation of boronic ester groups as ROS-responsive elements and the decoration of NP surfaces with erythrocyte membranes to camouflage the NPs from clearance after intravenous administration [140].

### 4.4. Biomaterials to Target Excitoxicity

Another line of research is the use of neuroprotective compounds to directly target excitoxicity. For example, the peptide NR2B9 (KLSSIESDV), derived from the carboxyl-terminal domain of the NMDAR NR2B subunit, is used to disrupt the interactions between the NMDA receptor with post-synaptic density protein 95 (PSD-95), to prevent the hyperproduction of NO and subsequent neuronal toxicity. Although this peptide is small (<1 KDa), its sequence contains polar and charged amino acids that represent the main obstacle to crossing the BBB or reaching the intracellular neuronal compartment. In a very interesting study, this peptide was delivered intranasally from PLGA NPs to circumvent the BBB [117]. The technological strategy used to increase the permeability of this material towards the brain was based on decorating the surface of the NPs with PEG to promote its penetration into mucus barrier. The authors also incorporated the wheat germ agglutinin (WGA), which binds to the N-acetyl-D-glucosamine and sialic acid present in the olfactory nasal epithelium and neuronal surface. This approach increased the content of NR2B9 in the brain relative to the intranasal administration of the free peptide. This more efficient delivery translated into a reduced infarct size and improvement of neurological deficits in a transient ischemic stroke model in rats [117]. Positive neuroprotective effects were also obtained with the same NR2B9C peptide delivered via dextran NPs decorated with the peptide CLEVSRKNC, which specifically targets neurons in ischemic areas [140]. These NPs were fully compatible in mice, and this strategy considerably reduced the infarct size, leading to the substantial functional recovery of rats with transient ischemia [140]. In another example, disruptors of GluN2B-PSD95-nNOS signalling were administrated from liposome-based NPs. This was the case for the experimental molecule, ZL006, which was delivered by an advanced soybean/lecithin/cholesterol-based liposome decorated with PEG, the CLEVSRKNC peptide, and the HAIYPRH peptide, which recognizes the transferring receptor of the brain’s endothelium [216]. In vitro, this engineered NP sustained the delivery of ZL006 over 30 h, with 50% of the molecule delivered in a time window of 3–5 h. In a transient focal ischemia rat model, these NPs reduced the infarct volume and improved neurological deficits, a fact ascribed to the neuroprotective effects of ZL006 and the special ability of these NPs to permeate the BBB and enter the brain during a temporal window of opportunity of at least 24 h [216]. Inhibitors of MMP-9, such as TIMP-1, have been used to reduce neuronal excitotoxicity and neuronal damage. This has been achieved even more efficiently through biomaterial-based delivery systems, such as PLGA-NPs, which promoted neuroprotection in hippocampal organotypic cultures and presented strong penetration through the BBB after decoration with polysorbate 80 [217,218]. Riluzole, an inhibitor of excitotoxicity, has been used in the treatment of Amyotrophic lateral sclerosis to increase patient survival. The mechanisms of action for Riluzole are not well understood, although their effects are variable, including the interruption of glutamatergic signalling and antagonizing the NMDA glutamate receptor or/and blocking the voltage-gated calcium and sodium channels, thereby promoting neuroprotection. To overcome the limited stability of Riluzole, this compound has been delivered through chitosan NPs formulated with N-isopropyl acrylamide and tween80 to favour the passage of these NPs through the BBB after intraperitoneal injection [219]. Interestingly, in an ischemia transient rat model, these engineered NPs reduced the extensive damage and improved neurological deficits in a dose-dependent manner. The positive effects of this therapy were due to the decreasing content of lipid peroxide and the increasing level of glutathione, as well as the activity of ROS detoxification enzymes, which translated into post-ischemia neuroprotection. However, although Riluzole has a reduced half-life, the effect of Riluzole administrated as a free molecule was not examined in this study, preventing to corroborate the additional advantages of encapsulating the drug in this specific NP formulation [219].

### 4.5. Other Strategies for Inducing Neuroprotection

Antagonists of the Nogo-66 receptors have been used to enhance neural plasticity and axonal rewiring after stroke or spinal cord injury [220]. In addition, Nogo-66 receptors are linked with the family of BCL-2, which regulate cell death, suggesting that suppressing Nogo receptor signaling might favor neuroprotection [221]. In this context, the intravenous injection of PLGA NPs decorated with chlorotoxin (to target MMP-2) and lexiscan (to transiently increase BBB permeability) allowed the delivery of the Nogo-66 receptor antagonist peptide, NEP1-40. This approach translated into a decrease in infarct volume and a better functional outcome in a transient ischemic model in mice [222]. Alternative strategies have been focused on preventing the disruption of the BBB and vascular networks in ischemic areas. This is the case for Cilostazol, which shows vasodilatation and platelet anti-aggregation properties, as well as anti-inflammatory and antioxidant potential. Significant neuroprotective effects related to a reduction in infarct size were observed when Cilostazol was delivered intravenously from methylcellulose NPs into a mouse brain ischemia/reperfusion model [223]. However, no significant differences were observed in the Cilostazol pharmacokinetics in plasma administrated as a free molecule or via methylcellulose NPs. Moreover, a direct comparison between the neuroprotective and functional outcomes of both methods of delivery was not performed [223].

One interesting approach is related to the delivery of genes and small interfering RNAs (siRNAs) coated in different polymers as a way to overexpress/silence specific genes that are abnormally down/up-regulated after injury [224]. The encapsulation of genes/siRNAs in advanced biomaterials constitutes an intelligent system for intracellular delivery to prevent the rapid enzymatic induced degradation of DNA/siRNAs in the extracellular space or bypass the low rate of transfection in terminally differentiated cells, such as neurons. Unlike the usual delivery of drugs/factors in the extracellular compartment, genes/siRNAs coated in NPs are directly internalized by specific cell populations. Based on their specific composition, these NPs might be used for gene/siRNA delivery and also for in vivo cell tracking with magnetic resonance imaging of the transplanted cells. For example, a peptidic-based micelle (R3V6) was stabilized in an aqueous solution with the anti-inflammatory and hydrophobic compound dexamethasone to deliver and overexpress the heme oxygenase-I (HO-I) in the brain [225]. This gene encodes an anti-oxidant enzyme that, when delivered from R3V6 NPs, reduced the infarct size in a rat model of transient ischemia; however, in this case, the treatment was applied one hour before ischemia reperfusion [225]. PAMAM dendrimers were coated with siRNAs to silence the expression of the High Mobility Group Box 1 factor, which is released from the damaged brain tissue leading to inflammation. The internalization of these dendrimers by neurons in vitro and in vivo decreased the post-ischemia neuronal mortality while reduced the infarct volume of stroke rats submitted to middle cerebral artery occlusion [226]. In other study, polyethylenimine superparamagnetic iron oxide NPs were used as carriers for silencing the hypoxia-inducible factor prolyl hydroxylase (PHID2) gene, with siRNAs delivered in endothelial progenitor cells [227]. In this study, the silencing of PHD2 caused an increase in the expression of Cxcr4, the receptor for Cxcl12. The axis of Cxcr4/Cxcl12 constitutes a signaling pathway that regulates stem cell mobilization, migration, and retention in different tissues, such as bone marrow and the brain [228]. This strategy enhanced the migration of endothelial progenitor cells intracardially injected towards the ischemic brain, thus stimulating endogenous vascularization and neurogenesis, as well as reducing the infarct size. All these effects were translated into the recovery of post-stroke sensorimotor deficits over a two-week study period [227]. Considering this particular signaling pathway, alternative approaches have employed pH-sensitive micelles to deliver Cxcl12 in the ischemic core whose acidic environment might release this factor as a result of charge repulsion [229]. In ischemic rats, the intravenous injection of urethane amino sulfamethazine-based NPs promoted angiogenesis and neurogenesis, although they did not exert any significant neuroprotective effect by reducing the infarct volume [229]. Although different neuroprotective drugs and recombinant factors might be loaded directly into NPs and hydrogels (such as VEGF, NFG, or Cxcl12), another interesting area is based on the encapsulation of undifferentiated or terminal cells in hydrogels and other biomaterial formats for the delivery of neuroprotective cytoquines and factors directly secreted from cells through exocytosis mechanisms. For further information on this very specific topic, a number of comprehensive reviews on the combination of therapeutic cells and biomaterials are given in the reference section [230,231,232,233].

## 5. Conclusions

Very few neuroprotective compounds have reached clinics after decades of preclinical research. This has been the case for Edaravone, an exclusive antioxidant and ROS scavenger compound that is currently being used to treat stroke patients in Japan. However, the actual context of such drugs problematizes the exploration of therapeutic solutions that are able to overcome the unexpected lack of clinical translatability, despite successful animal trials. In recent years, considerable progress has been made in the identification of advanced biomaterial formulations for the controlled delivery of neuroprotective molecules. The vast majority of studies have demonstrated that these formulations deliver neuroprotective molecules in the brain at therapeutic doses for prolonged time periods, achieving greater therapeutic responses than those obtained with the simple administration of a free molecule. Different administration routes have been tested. While hydrogels have preferably been used for intracranial applications (infarcted cavity, epicortical, and peri-lesional regions), nanoparticles have shown greater versatility of use, as they can be administered through almost all known routes. Among the latest trends, intranasal delivery is very promising to avoid invasive intracranial procedures by bypassing the blood–brain barrier and avoiding the rapid decay and degradation observed with molecules administrated systemically (for example, intravenously). However, it would be necessary to move the actual research on this area in several directions. Firstly, there is great versatility and heterogeneity between studies and a lack of connections between them. In the majority of trials, no comparisons have been done between different material formulations in similar stroke models to determine which of them have better performance rates. In addition, for a given material, we lack a rational comparison between the routes and times of administration. The inherent complexity of biomaterial-based studies covering several disciplines (physical, chemistry, and biology) and the difficulty and variability of animal trials have likely contributed to this scenario. A consequence of this is the lack of consensus on the best route and optimal window of administration. Secondly, the molecular, cellular, and functional mechanisms of behavioral improvements in response to biomaterial-based treatments are also largely unknown. Thirdly, connections with clinics have failed. Certainly, none of the known stroke animal models are a perfect representation of human disease, but most animal trials have been performed in ischemia/reperfusion models. However, ischemia and reperfusion do not reflect the most frequent forms of stroke in humans, since the majority of stroke patients are refractory to treatment or are outside of the therapeutic windows, as indicated by the Stroke Academic Industry Roundtable (STAIR). The most invasive administration routes (for example, intracerebral), although very effective in animals and a valuable proof of concept for biomaterial-based therapeutics, will probably be of little value in a clinical context when treating real patients. Although most of the tested nanoparticles and hydrogels appear to be harmless in animal trials, there is an unknown horizon with respect to their safety and tolerability in the human brain. To the best of our knowledge, no single material has reached the clinical setting with the aim to control the delivery of therapeutic molecules in the brain. An exception is Gliadel, a polyanhydride polymer that is used against glioblastoma multiforme by delivering the anti-tumoral drug carmustine [234]. The clinical ineffectiveness of neuroprotective compounds in parallel to the need for large/expensive trials compared with other biomedical areas, has probably conditioned/restricted the use of biomaterials in clinical stroke. Given the realistic impossibility of obtaining extensive clinical information on this matter, significant steps in the screening of biomaterials/drugs for clinical use could be achieved using relatively recent technologies, such as organoids, to model the human blood–brain barrier and the structured neurovascular tissue.

## Figures and Tables

**Table 1:** Examples of in vivo studies with the aim to neuroprotect and functionally recover the damaged brain using nanoparticle- and hydrogel-based biomaterials.

Stroke Model, Species	Therapeutic Molecule, Main Properties	Biomaterial and Format, Administration Route, Time Window of Application	Main Therapeutic Effects	References
MCAO (permanent), rat	Superoxide dismutase/Catalase, anti-oxidant enzymes	PGA NPs, intravenous injection, ~ 1 min post-stroke	Reduction of infarct volume	[49]
MCAO (transient), mouse	BDNF, neuroprotective, neurogenic, and regenerative factor	PEG/PGA NPs, intravenous injection, 3–24 h post-stroke	Reduction in cerebral tissue loss	[134]
MCAO (transient), rat	Osteopontin, anti-inflammatory, anti-apoptotic, and neurogenic roles	Gelatin microspheres, intrastriatal delivery, 1–12 h post-stroke	Reduced infarct volume	[199]
Photothrombotic stroke model (permanent), mouse	Edaravone, antioxidant compound/ROS scavenger	PEG-PLA micelles, intravenous injection, 6 h post-stroke	Higher efficiency of Edaravone delivery. Reduction of ROS, decreasing of ischemic area that leads to behavioural improvement	[152]
Endothelin-1 model, rat	Cyclosporin-A, immunosuppressant and anti-inflammatory compound. Neurogenic and regenerative properties	PLGA microparticles encapsulated in hyaluronan and methylcellulose hydrogels, epicortical delivery, immediately after entothelin-1 injection	Sustained delivery of Cyclosporin-A for 14 days. Decreasing infarct volume. Neuroprotective effect even with the hydrogel formulation alone.	[153]
Endothelin-1 model, mouse	EGF/erythropoietin, stimulators of neural precursors formation and neuroprotective properties	PLGA-PEG NPs encapsulated in hyaluronan and methylcellulose hydrogels, epicortical administration, 4 days post-stroke	Attenuation of injury response and cell death. Reduction of infarct cavity. Increasing content of mature neurons in damage and peri-lesional areas	[154]
Focal aspiration brain injury model, rat	Neurotrophin-3, neurogenic, angiogenic, and anti-inflammatory factor	Chitosan particles, intracerebral delivery (lesion cavity, cortex), immediately after tissue aspiration	Reduction of inflammation (peripheral leukocytes and microglia)	[169]
Hypoxia-ischemia exposure model, neonatal rat	Erythropoietin, enhances oxygen delivery, neurogenesis, anti-oxidant properties (decreases ROS formation)	PLGA NPs, intraperitoneal, 1, 24, and 48 h after hypoxia	Reduction of infarct volume and improvement of behavioral deficits with lower doses than the administration of free Erythropoietin	[196]
MCAO (transient), rat	Osteopontin, anti-inflammatory, anti-apoptotic, and neurogenic roles	Gelatin microspheres, intranasal, 6 h post-stroke	Reduction of infarct volume	[200]
MCAO (transient), rat	Superoxide dismutase, anti-oxidant enzyme	PLGA NPs, arterial (intracarotid), immediately after reperfusion (1 h post-ischemia)	Reduction of infarct volume leading to functional recovery	[203]
MCAO (transient), mouse	Superoxide dismutase, anti-oxidant enzyme	Polyion-cationic complexes-PEG, intravenous injection, immediately after reperfusion (1 h post-ischemia)	Reduction of infarct volume (no effect with superoxide dismutase alone)	[205]
MCAO (transient), rat	Thymoquinone, anti-inflammatory and anti-oxidant factor	PLGA-Chitosan NPs, intranasal, administration for 12 days: from -5 (pre-stroke) to 7 (post-stroke) days	Reduction of infarct volume and significant motor improvement associated with stronger scavenging and anti-oxidant capacity	[208]
Control (healthy), rat	Venlafaxine, antidepressant molecule	Chitosan NPs, intranasal delivery	Efficient delivery of Venlafaxine into the brain via the intranasal route in comparison with the intranasal or intravenous delivery of free Venlafaxine	[209]
MCAO (transient), rat	Acetyl-11-keto-β-boswellic acid, anti-oxidant and anti-inflammatory effects	Chitosan NPs, intravenous injection, 1 h after reperfusion (2 h post-ischemia)	Higher rates of delivery in the brain with respect to the administration of the free molecule. Reduction of infarct volume that leads to behavioral improvements. Positive outcomes related with decreasing oxidative stress/inflammation and reduced neuronal mortality post-ischemia	[210]
MCAO (transient), rat	Fenofibrate, activator of Peroxisome Proliferator Activated Receptors (anti-inflammatory and anti-oxidative receptors)	PLGA microparticles, intracerebral administration, 24 h before ischemia	Moderate (non-significant) decrease of infarct volume with respect to the effect of free Fenofibrate. Evolution of infarct volume on untreated MCAO animals is non-determined	[211]
MCAO (transient), rat	ONO-1301, prostacyclin agonist with thromboxane synthase inhibitory activity	PLGA microspheres, subcutaneous injection, immediately after MCAO	Significant reduction in the infarct volume and cerebral edema. Oral administration of the free molecule produced similar neuroprotective efficacy, although repetitive doses were needed.	[214]
Global cerebral (transient), rat	Curcumin, antioxidant and anti-inflammatory effects	Polysorbate 80/lecithin-based NPs, oral administration, starting 5 days before ischemia for 3 days	Motor and cognitive improvement. No positive effect with the free molecule	[215]
MCAO (transient), rat	NR2B9, prevents the excitotoxocity and hyperproduction of NO by disrupting NMDA-PSD-95 signaling	PLGA-PEG nanoparticles decorated with wheat germ agglutinin (WGA) to target olfactory epithelium receptors, intranasal delivery, immediately after reperfusion (2 h post-ischemia)	Increasing content of NR2B9 in the brain with respect to the intranasal administration of free molecule. Significant reduction in infarct size and better neurological improvement	[117]
MCAO (transient), rat	NR2B9, prevents the excitotoxocity and hyperproduction of NO by disrupting NMDA-PSD-95 signaling	Dextran NPs decorated with ROS-responsive boronic ester and red blood shell membrane, intravenous injection, immediately after reperfusion (2 h post-ischemia)	NR2B9: Increasing time in circulation and highly efficient to target NR2B9 in the brain. Significant reduction in infarct size and better neurological outcome	[140]
MCAO (transient), rat	ZL006, prevents the excitotoxocity and hyperproduction of NO by disrupting NMDA-PSD-95 signaling	Soybean-lecithin-cholesterol-based liposome-PEG decorated with peptides against transferring receptor of the brain endothelium, intravenous injection	Reduction of infarct volume. Behavioural improvement	[216]
MCAO (transient), rat	Riluzole, variable effects antagonizing NMDA receptors or/and blocking voltage-gated Calcium/sodium channels	Chitosan NPs formulated with N-isopropyl acrylamide/tween80, intraperitoneal injection, 1 h post-ischemia	Decreasing content of lipid peroxide and increasing levels of glutathione and ROS detoxification enzymes. Reduction of lesion and improvement of behavioral deficits	[219]
MCAO (transient), mouse	Cilostazol, vasodilatating, anti-inflammatory with anti-oxidant properties	Zirconia/Methylcellulose NPs, intravenous delivery, 3 h after reperfusion (5 h post-ischemia)	Reduction of infarct size	[223]
MCAO (transient), mouse	NEP1-40, antagonist of the Nogo-66 receptor,. enhances neural plasticity and suppresses cell death after injury	PLGA NPs decorated with chlorotoxin to target MMP-2 and lexiscan to transiently increase the BBB permeability, intravenous injection, 3 doses (0, 24, and 48 h after MCAO)	Reduction of infarct volume and better neurological outcome	[222]
MCAO (transient), rat	Heme oxygenase-1, anti-oxidant enzyme	R3V6-peptide -based micelles stabilized with Dexamethasone, intracerebral injection (striatum), 1 h before ischemia	Reduction of infarct volume	[225]
MCAO (transient), rat	siRNA against HMGB1, (HMGB1 is a pro-inflammatory signal)	PAMAM-based dendrimers, intracerebral injection (cortex). 6–24 h before ischemia	Reduction of neuronal mortality and infarct volume	[226]
Photothrombotic stroke model (permanent), mouse	siRNA to silence PHID2, (PHD2 is a factor involved in the up-regulation of genes related to a cellular response to hypoxia)	Polyethylenimine superparamagnetic iron oxide NPs delivered from endothelial progenitor cells, intra-cardiac injection, 24 h after stroke	Significant reduction of infarct volume at 7 days after treatment. Stimulation of endogenous vascularization and neurogenesis. Significant Improvement of behavioral deficits (at 2 weeks after treatment)	[227]
MCAO (permanent), rat	Cxcl12, a chemoattractant and neuroprotective molecule	pH-responsive copolymer poly (urethane amino sulfamethazine) (PUASM) micelles, intracerebral delivery, 24 h after ischemia	Significant angiogenesis and neurogenesis, lack of a neuroprotective effect	[229]
MCAO (transient), rat	VEGF, an angiogenic and neuroprotectant molecule	Alginate-based hydrogels, intracerebral injection (striatum), 15 min before ischemia	Higher content of VEGF administrated in the striatum than delivery of the free molecule. Behavioral recovery and significant reduction of infarct volume	[168]
Focal aspiration brain injury model, rat	Neurotrophin-3, neuroprotective, neurogenic, and anti-inflammatory factor	Chitosan particles, intracerebral administration (cortex), immediately after injury	Increasing neurogenesis and synaptogenesis, anti-inflammatory effects, and behavioral improvement	[169]
Endothelin-1 model, rat	BDNF, a neuroprotector molecule and inductor of plasticity	PLGA NPs dispersed on the hyaluronan and methylcellulose hydrogels, epi-cortical administration (brain surface), immediately after stroke	Significant behavioral recovery and reduced cortical lesions (these positive effects were seen even with the biomaterial alone)	[170]
Endothelin-1 model, mouse	Dual delivery of EGF and EPO, neurogenic, anti-inflammatory, and neuroprotective factors	PLGA NPs dispersed on the hyaluronan and methylcellulose hydrogels, epi-cortical administration (brain surface), 4 days after stroke	Increasing neurogenesis and reduction of injury response, inflammation and cell death (attenuation of inflammation and injury were also observed, even with the biomaterial alone)	[154]
MCAO (transient), hyperglycemic rat	Carbon-HCCs, antioxidant potential	Carbon-PEG NPs, intravenous administration, two doses (immediately before reperfusion and 2 h after reperfusion)	Reduction in infarct size and behavioral improvement	[183]
MCAO (transient), rat	Selenium, modulator of neurogenesis with anti-oxidant properties	Selenium-PEG NPs decorated with Anti-transferrin receptor antibody, intraperitoneal injection, 1 h before stroke	Reduction in infarct size, high levels of myelination, neural, and axonal density. Behavioral improvement. No toxicity concerns at therapeutic doses	[184]
MCAO (permanent), mouse	Hyaluronic acid hydrogel (porous), promotes cell neural stem cell infiltration and reduces inflammation	MAP-HA-based hydrogels, intracerebral delivery (stroke cavity), 5 days post-stroke	Reduction of inflammation and astrogliosis, increasing neurogenesis and angiogenesis. Remarkable neural progenitor cell migration to lesional and peri-lesional areas	[161]
MCAO (transient), rat	Porcine urinary bladder ECM, promotes cell neural stem cell infiltration and reduces inflammation	Porcine urinary bladder ECM hydrogels, intracerebral administration (stroke cavity), 14 days post-stroke	Increasing microglia polarization towards anti-inflammatory phenotypes	[181]
MCAO (transient), macaque monkey	Hemoglobin, oxygen carrier supporting tissue oxygenation	Phosphatidylcholine-cholesterol-liposome decorated with PEG, intravenous injection, 5 min after ischemia	Reduction of infarct area, behavioral improvement	[235,236]
MCAO (transient), macaque monkey	BDNF, neuroprotective, neurogenic, and regenerative factor	HA-PEG based hydrogel, intracerebral administration (stroke cavity), 3 months post-stroke	Significant increase of BDNF content in peri-infarct tissue in relation to the delivery of free BDNF	[156]
Control (healthy), St. Kitts green monkey	No therapeutic compound	PEG-PLA-based hydrogel, intracerebral injection (cortex and striatum)	Moderate inflammation and astrogliosis. Full degradation of material four months after implantation	[155]

Table abbreviations; NPs: nanoparticles; PGA: poly(glycolic acid); PLGA: poly(Lactide-co-Glycolide); PLA: Poly (Lactic Acid); PEG: Polyethylene glycol; HA: hyaluronic acid; NO: Nitric oxide; MCAO: middle cerebral artery occlusion; BNDF: brain-derived neurotrophic factor; EGF: Epidermal Growth Factor; VEGF: Vascular endothelial growth factor; EPO: Erythropoietin; ROS: Reactive oxygen species; NMDA: N-methyl-D-aspartate receptor; PSD-95: postsynaptic density protein 95; WGA: Wheat germ agglutinin; MMP-2: Matrix Metallopeptidase 2; BBB: blood–brain barrier; HMGB1: high mobility group box 1; PAMAM: Polyamidoamine; siRNA: Small interfering RNA; Cxcl12: stromal cell-derived factor 1 or C-X-C motif chemokine 12; PHD2: hydroxylase domain protein 2; ECM: extracellular matrix.

## References

[1] Donnan G.A., Fisher M., Macleod M., Davis S.M. (2008). Stroke. Lancet.

[2] Enderby P., Pandyan A., Bowen A., Hearnden D., Ashburn A., Conroy P., Logan P., Thompson C., Winter J. (2017). Accessing rehabilitation after stroke—A guessing game?. Disabil. Rehabil..

[3] Moon S.K., Alaverdashvili M., Cross A.R., Whishaw I.Q. (2009). Both compensation and recovery of skilled reaching following small photothrombotic stroke to motor cortex in the rat. Exp. Neurol..

[4] Broderick J.P., Palesch Y.Y., Demchuk A.M., Yeatts S.D., Khatri P., Hill M.D., Jauch E.C., Jovin T.G., Yan B., Silver F.L. (2013). Endovascular therapy after intravenous t-PA versus t-PA alone for stroke. N. Engl. J. Med..

[5] Saver J.L., Goyal M., Bonafe A., Diener H.C., Levy E.I., Pereira V.M., Albers G.W., Cognard C., Cohen D.J., Hacke W. (2015). Stent-retriever thrombectomy after intravenous t-PA vs. t-PA alone in stroke. N. Engl. J. Med..

[6] Kleindorfer D., Kissela B., Schneider A., Woo D., Khoury J., Miller R., Alwell K., Gebel J., Szaflarski J., Pancioli A. (2004). Eligibility for recombinant tissue plasminogen activator in acute ischemic stroke: A population-based study. Stroke.

[7] Pantano P., Caramia F., Bozzao L., Dieler C., von Kummer R. (1999). Delayed increase in infarct volume after cerebral ischemia: Correlations with thrombolytic treatment and clinical outcome. Stroke.

[8] Shi K., Tian D.C., Li Z.G., Ducruet A.F., Lawton M.T., Shi F.D. (2019). Global brain inflammation in stroke. Lancet Neurol..

[9] Zhao S.C., Ma L.S., Chu Z.H., Xu H., Wu W.Q., Liu F. (2017). Regulation of microglial activation in stroke. Acta. Pharmacol. Sin..

[10] Chamorro A., Dirnagl U., Urra X., Planas A.M. (2016). Neuroprotection in acute stroke: Targeting excitotoxicity, oxidative and nitrosative stress, and inflammation. Lancet Neurol..

[11] Chen H., Yoshioka H., Kim G.S., Jung J.E., Okami N., Sakata H., Maier C.M., Narasimhan P., Goeders C.E., Chan P.H. (2011). Oxidative stress in ischemic brain damage: Mechanisms of cell death and potential molecular targets for neuroprotection. Antioxid. Redox Signal..

[12] Lambertsen K.L., Finsen B., Clausen B.H. (2019). Post-stroke inflammation-target or tool for therapy?. Acta. Neuropathol..

[13] Ayata C. (2013). Spreading depression and neurovascular coupling. Stroke.

[14] Hill M.D., Goyal M., Menon B.K., Nogueira R.G., McTaggart R.A., Demchuk A.M., Poppe A.Y., Buck B.H., Field T.S., Dowlatshahi D. (2020). Efficacy and safety of nerinetide for the treatment of acute ischaemic stroke (ESCAPE-NA1): A multicentre, double-blind, randomised controlled trial. Lancet.

[15] Matsumoto S., Murozono M., Kanazawa M., Nara T., Ozawa T., Watanabe Y. (2018). Edaravone and cyclosporine A as neuroprotective agents for acute ischemic stroke. Acute Med. Surg..

[16] (2006). Neuroprotection: The end of an era?. Lancet.

[17] Shi L., Rocha M., Leak R.K., Zhao J., Bhatia T.N., Mu H., Wei Z., Yu F., Weiner S.L., Ma F. (2018). A new era for stroke therapy: Integrating neurovascular protection with optimal reperfusion. J. Cereb. Blood Flow Metab..

[18] Louveau A., Da Mesquita S., Kipnis J. (2016). Lymphatics in Neurological Disorders: A Neuro-Lympho-Vascular Component of Multiple Sclerosis and Alzheimer’s Disease?. Neuron.

[19] Cornford E.M., Hyman S. (2005). Localization of brain endothelial luminal and abluminal transporters with immunogold electron microscopy. NeuroRx.

[20] De Bock M., Van Haver V., Vandenbroucke R.E., Decrock E., Wang N., Leybaert L. (2016). Into rather unexplored terrain-transcellular transport across the blood-brain barrier. Glia.

[21] Illum L. (2004). Is nose-to-brain transport of drugs in man a reality?. J. Pharm. Pharmacol..

[22] Keum S., Marchuk D.A. (2009). A locus mapping to mouse chromosome 7 determines infarct volume in a mouse model of ischemic stroke. Circ. Cardiovasc Genet..

[23] Becker K.J. (2016). Strain-Related Differences in the Immune Response: Relevance to Human Stroke. Transl. Stroke Res..

[24] Upadhyay R.K. (2014). Drug delivery systems, CNS protection, and the blood brain barrier. Biomed. Res. Int..

[25] Dong X. (2018). Current Strategies for Brain Drug Delivery. Theranostics.

[26] Begley D.J., Brightman M.W. (2003). Structural and functional aspects of the blood-brain barrier. Prog. Drug Res..

[27] Chen B., Friedman B., Cheng Q., Tsai P., Schim E., Kleinfeld D., Lyden P.D. (2009). Severe blood-brain barrier disruption and surrounding tissue injury. Stroke.

[28] Neumann-Haefelin T., Kastrup A., de Crespigny A., Yenari M.A., Ringer T., Sun G.H., Moseley M.E. (2000). Serial MRI after transient focal cerebral ischemia in rats: Dynamics of tissue injury, blood-brain barrier damage, and edema formation. Stroke.

[29] Albers G.W. (2018). Late Window Paradox. Stroke.

[30] Bernhardt J., Hayward K.S., Kwakkel G., Ward N.S., Wolf S.L., Borschmann K., Krakauer J.W., Boyd L.A., Carmichael S.T., Corbett D. (2017). Agreed definitions and a shared vision for new standards in stroke recovery research: The Stroke Recovery and Rehabilitation Roundtable taskforce. Int. J. Stroke.

[31] Dobkin B.H., Carmichael S.T. (2016). The Specific Requirements of Neural Repair Trials for Stroke. Neurorehabil. Neural Repair.

[32] Kuhlmann C.R., Gerigk M., Bender B., Closhen D., Lessmann V., Luhmann H.J. (2008). Fluvastatin prevents glutamate-induced blood-brain-barrier disruption in vitro. Life Sci.

[33] Yang G., Chan P.H., Chen S.F., Babuna O.A., Simon R.P., Weinstein P.R. (1994). Reduction of vasogenic edema and infarction by MK-801 in rats after temporary focal cerebral ischemia. Neurosurgery.

[34] Bordi F., Pietra C., Ziviani L., Reggiani A. (1997). The glycine antagonist GV150526 protects somatosensory evoked potentials and reduces the infarct area in the MCAo model of focal ischemia in the rat. Exp. Neurol..

[35] Pearlstein R.D., Beirne J.P., Massey G.W., Warner D.S. (1998). Neuroprotective effects of NMDA receptor glycine recognition site antagonism: Dependence on glycine concentration. J. Neurochem.

[36] Hoyte L., Barber P.A., Buchan A.M., Hill M.D. (2004). The rise and fall of NMDA antagonists for ischemic stroke. Curr. Mol. Med..

[37] Lai T.W., Zhang S., Wang Y.T. (2014). Excitotoxicity and stroke: Identifying novel targets for neuroprotection. Prog. Neurobiol..

[38] Neuhaus A.A., Couch Y., Hadley G., Buchan A.M. (2017). Neuroprotection in stroke: The importance of collaboration and reproducibility. Brain.

[39] Bratane B.T., Cui H., Cook D.J., Bouley J., Tymianski M., Fisher M. (2011). Neuroprotection by freezing ischemic penumbra evolution without cerebral blood flow augmentation with a postsynaptic density-95 protein inhibitor. Stroke.

[40] Cook D.J., Teves L., Tymianski M. (2012). Treatment of stroke with a PSD-95 inhibitor in the gyrencephalic primate brain. Nature.

[41] Hill M.D., Martin R.H., Mikulis D., Wong J.H., Silver F.L., Terbrugge K.G., Milot G., Clark W.M., Macdonald R.L., Kelly M.E. (2012). Safety and efficacy of NA-1 in patients with iatrogenic stroke after endovascular aneurysm repair (ENACT): A phase 2, randomised, double-blind, placebo-controlled trial. Lancet Neurol..

[42] Sims N.R., Muyderman H. (2010). Mitochondria, oxidative metabolism and cell death in stroke. Biochim Biophys Acta..

[43] Carbone F., Teixeira P.C., Braunersreuther V., Mach F., Vuilleumier N., Montecucco F. (2015). Pathophysiology and Treatments of Oxidative Injury in Ischemic Stroke: Focus on the Phagocytic NADPH Oxidase 2. Antioxid. Redox Signal..

[44] Zalba G., Fortuno A., San Jose G., Moreno M.U., Beloqui O., Diez J. (2007). Oxidative stress, endothelial dysfunction and cerebrovascular disease. Cerebrovasc. Dis..

[45] Pozo-Rodrigalvarez A., Gradillas A., Serrano J., Fernandez A.P., Martinez-Murillo R., Perez-Castells J. (2012). New synthesis and promising neuroprotective role in experimental ischemic stroke of ONO-1714. Eur. J. Med. Chem..

[46] Murakami K., Kondo T., Kawase M., Li Y., Sato S., Chen S.F., Chan P.H. (1998). Mitochondrial susceptibility to oxidative stress exacerbates cerebral infarction that follows permanent focal cerebral ischemia in mutant mice with manganese superoxide dismutase deficiency. J. Neurosci.

[47] Moro M.A., Almeida A., Bolanos J.P., Lizasoain I. (2005). Mitochondrial respiratory chain and free radical generation in stroke. Free Radic Biol Med..

[48] Kinouchi H., Epstein C.J., Mizui T., Carlson E., Chen S.F., Chan P.H. (1991). Attenuation of focal cerebral ischemic injury in transgenic mice overexpressing CuZn superoxide dismutase. Proc. Natl. Acad. Sci. USA.

[49] Liu T.H., Beckman J.S., Freeman B.A., Hogan E.L., Hsu C.Y. (1989). Polyethylene glycol-conjugated superoxide dismutase and catalase reduce ischemic brain injury. Am. J. Physiol..

[50] Ren Y., Wei B., Song X., An N., Zhou Y., Jin X., Zhang Y. (2015). Edaravone’s free radical scavenging mechanisms of neuroprotection against cerebral ischemia: Review of the literature. Int. J. Neurosci..

[51] Kuroda S., Tsuchidate R., Smith M.L., Maples K.R., Siesjo B.K. (1999). Neuroprotective effects of a novel nitrone, NXY-059, after transient focal cerebral ischemia in the rat. J. Cereb. Blood Flow Metab..

[52] Marshall J.W., Cummings R.M., Bowes L.J., Ridley R.M., Green A.R. (2003). Functional and histological evidence for the protective effect of NXY-059 in a primate model of stroke when given 4 h after occlusion. Stroke.

[53] Shuaib A., Lees K.R., Lyden P., Grotta J., Davalos A., Davis S.M., Diener H.C., Ashwood T., Wasiewski W.W., Emeribe U. (2007). NXY-059 for the treatment of acute ischemic stroke. N. Engl. J. Med..

[54] Chamorro A., Amaro S., Castellanos M., Segura T., Arenillas J., Marti-Fabregas J., Gallego J., Krupinski J., Gomis M., Canovas D. (2014). Safety and efficacy of uric acid in patients with acute stroke (URICO-ICTUS): A randomised, double-blind phase 2b/3 trial. Lancet Neurol..

[55] Turner R.C., Naser Z.J., Lucke-Wold B.P., Logsdon A.F., Vangilder R.L., Matsumoto R.R., Huber J.D., Rosen C.L. (2017). Single low-dose lipopolysaccharide preconditioning: Neuroprotective against axonal injury and modulates glial cells. Neuroimmunol. Neuroinflamm..

[56] Jin R., Yang G., Li G. (2010). Inflammatory mechanisms in ischemic stroke: Role of inflammatory cells. J. Leukoc Biol..

[57] Perego C., Fumagalli S., De Simoni M.G. (2011). Temporal pattern of expression and colocalization of microglia/macrophage phenotype markers following brain ischemic injury in mice. J. Neuroinflammation.

[58] Orihuela R., McPherson C.A., Harry G.J. (2016). Microglial M1/M2 polarization and metabolic states. Br. J. Pharmacol..

[59] Wang J., Xing H., Wan L., Jiang X., Wang C., Wu Y. (2018). Treatment targets for M2 microglia polarization in ischemic stroke. Biomed. Pharmacother..

[60] Jassam Y.N., Izzy S., Whalen M., McGavern D.B., El Khoury J. (2017). Neuroimmunology of Traumatic Brain Injury: Time for a Paradigm Shift. Neuron.

[61] Asahi M., Asahi K., Jung J.C., del Zoppo G.J., Fini M.E., Lo E.H. (2000). Role for matrix metalloproteinase 9 after focal cerebral ischemia: Effects of gene knockout and enzyme inhibition with BB-94. J. Cereb. Blood Flow Metab..

[62] Asahi M., Wang X., Mori T., Sumii T., Jung J.C., Moskowitz M.A., Fini M.E., Lo E.H. (2001). Effects of matrix metalloproteinase-9 gene knock-out on the proteolysis of blood-brain barrier and white matter components after cerebral ischemia. J. Neurosci..

[63] Chaturvedi M., Kaczmarek L. (2014). Mmp-9 inhibition: A therapeutic strategy in ischemic stroke. Mol. Neurobiol.

[64] Wang H., Song G., Chuang H., Chiu C., Abdelmaksoud A., Ye Y., Zhao L. (2018). Portrait of glial scar in neurological diseases. Int. J. Immunopathol. Pharmacol..

[65] Zou J., Wang Y.X., Dou F.F., Lu H.Z., Ma Z.W., Lu P.H., Xu X.M. (2010). Glutamine synthetase down-regulation reduces astrocyte protection against glutamate excitotoxicity to neurons. Neurochem. Int..

[66] Dubbelaar M.L., Kracht L., Eggen B.J.L., Boddeke E. (2018). The Kaleidoscope of Microglial Phenotypes. Front. Immunol..

[67] He Y., Ma X., Li D., Hao J. (2017). Thiamet G mediates neuroprotection in experimental stroke by modulating microglia/macrophage polarization and inhibiting NF-kappaB p65 signaling. J. Cereb. Blood Flow Metab..

[68] Shao Y., Deng T., Zhang T., Li P., Wang Y. (2015). FAM19A3, a novel secreted protein, modulates the microglia/macrophage polarization dynamics and ameliorates cerebral ischemia. FEBS Lett..

[69] Jickling G.C., Liu D., Ander B.P., Stamova B., Zhan X., Sharp F.R. (2015). Targeting neutrophils in ischemic stroke: Translational insights from experimental studies. J. Cereb. Blood Flow Metab..

[70] Lavine S.D., Hofman F.M., Zlokovic B.V. (1998). Circulating antibody against tumor necrosis factor-alpha protects rat brain from reperfusion injury. J. Cereb. Blood Flow Metab..

[71] Gary D.S., Bruce-Keller A.J., Kindy M.S., Mattson M.P. (1998). Ischemic and excitotoxic brain injury is enhanced in mice lacking the p55 tumor necrosis factor receptor. J. Cereb. Blood Flow Metab..

[72] Lambertsen K.L., Clausen B.H., Babcock A.A., Gregersen R., Fenger C., Nielsen H.H., Haugaard L.S., Wirenfeldt M., Nielsen M., Dagnaes-Hansen F. (2009). Microglia protect neurons against ischemia by synthesis of tumor necrosis factor. J. Neurosci..

[73] Rothwell N. (2003). Interleukin-1 and neuronal injury: Mechanisms, modification, and therapeutic potential. Brain Behav. Immun..

[74] Villa P., Triulzi S., Cavalieri B., Di Bitondo R., Bertini R., Barbera S., Bigini P., Mennini T., Gelosa P., Tremoli E. (2007). The interleukin-8 (IL-8/CXCL8) receptor inhibitor reparixin improves neurological deficits and reduces long-term inflammation in permanent and transient cerebral ischemia in rats. Mol. Med..

[75] Brait V.H., Rivera J., Broughton B.R., Lee S., Drummond G.R., Sobey C.G. (2011). Chemokine-related gene expression in the brain following ischemic stroke: No role for CXCR2 in outcome. Brain Res..

[76] Dimitrijevic O.B., Stamatovic S.M., Keep R.F., Andjelkovic A.V. (2007). Absence of the chemokine receptor CCR2 protects against cerebral ischemia/reperfusion injury in mice. Stroke.

[77] Chu H.X., Broughton B.R., Kim H.A., Lee S., Drummond G.R., Sobey C.G. (2015). Evidence That Ly6C(hi) Monocytes are Protective in Acute Ischemic Stroke by Promoting M2 Macrophage Polarization. Stroke.

[78] Werner Y., Mass E., Ashok Kumar P., Ulas T., Handler K., Horne A., Klee K., Lupp A., Schutz D., Saaber F. (2020). Cxcr4 distinguishes HSC-derived monocytes from microglia and reveals monocyte immune responses to experimental stroke. Nat. Neurosci..

[79] Dreier J.P., Fabricius M., Ayata C., Sakowitz O.W., William Shuttleworth C., Dohmen C., Graf R., Vajkoczy P., Helbok R., Suzuki M. (2017). Recording, analysis, and interpretation of spreading depolarizations in neurointensive care: Review and recommendations of the COSBID research group. J. Cereb. Blood Flow Metab..

[80] Nakamura H., Strong A.J., Dohmen C., Sakowitz O.W., Vollmar S., Sue M., Kracht L., Hashemi P., Bhatia R., Yoshimine T. (2010). Spreading depolarizations cycle around and enlarge focal ischaemic brain lesions. Brain.

[81] Klass A., Sanchez-Porras R., Santos E. (2018). Systematic review of the pharmacological agents that have been tested against spreading depolarizations. J. Cereb. Blood Flow Metab..

[82] Sakowitz O.W., Kiening K.L., Krajewski K.L., Sarrafzadeh A.S., Fabricius M., Strong A.J., Unterberg A.W., Dreier J.P. (2009). Preliminary evidence that ketamine inhibits spreading depolarizations in acute human brain injury. Stroke.

[83] Barios J.A., Pisarchyk L., Fernandez-Garcia L., Barrio L.C., Ramos M., Martinez-Murillo R., Gonzalez-Nieto D. (2016). Long-term dynamics of somatosensory activity in a stroke model of distal middle cerebral artery oclussion. J. Cereb. Blood Flow Metab..

[84] Fernandez-Garcia L., Perez-Rigueiro J., Martinez-Murillo R., Panetsos F., Ramos M., Guinea G.V., Gonzalez-Nieto D. (2018). Cortical Reshaping and Functional Recovery Induced by Silk Fibroin Hydrogels-Encapsulated Stem Cells Implanted in Stroke Animals. Front. Cell Neurosci..

[85] Wang Y., Zhao Z., Rege S.V., Wang M., Si G., Zhou Y., Wang S., Griffin J.H., Goldman S.A., Zlokovic B.V. (2016). 3K3A-activated protein C stimulates postischemic neuronal repair by human neural stem cells in mice. Nat. Med..

[86] Huebsch N., Mooney D.J. (2009). Inspiration and application in the evolution of biomaterials. Nature.

[87] Green J.J., Elisseeff J.H. (2016). Mimicking biological functionality with polymers for biomedical applications. Nature.

[88] Hubbell J.A., Thomas S.N., Swartz M.A. (2009). Materials engineering for immunomodulation. Nature.

[89] Laflamme M.A., Murry C.E. (2011). Heart regeneration. Nature.

[90] Khader B.A., Towler M.R. (2016). Materials and techniques used in cranioplasty fixation: A review. Mater. Sci. Eng. C Mater. Biol. Appl..

[91] Still M., Kane A., Roux A., Zanello M., Dezamis E., Parraga E., Sauvageon X., Meder J.F., Pallud J. (2018). Independent Factors Affecting Postoperative Complication Rates After Custom-Made Porous Hydroxyapatite Cranioplasty: A Single-Center Review of 109 Cases. World Neurosurg..

[92] Taschner C.A., Chapot R., Costalat V., Machi P., Courtheoux P., Barreau X., Berge J., Pierot L., Kadziolka K., Jean B. (2018). Second-Generation Hydrogel Coils for the Endovascular Treatment of Intracranial Aneurysms: A Randomized Controlled Trial. Stroke.

[93] Mehta R.I. (2016). Polymer-induced central nervous system complications following vascular procedures: Spectrum of iatrogenic injuries and review of outcomes. Hum. Pathol..

[94] Sugawara T., Fujimura M., Noshita N., Kim G.W., Saito A., Hayashi T., Narasimhan P., Maier C.M., Chan P.H. (2004). Neuronal death/survival signaling pathways in cerebral ischemia. NeuroRx.

[95] Xiong X.Y., Liu L., Yang Q.W. (2016). Functions and mechanisms of microglia/macrophages in neuroinflammation and neurogenesis after stroke. Prog. Neurobiol..

[96] Ruscher K., Kuric E., Liu Y., Walter H.L., Issazadeh-Navikas S., Englund E., Wieloch T. (2013). Inhibition of CXCL12 signaling attenuates the postischemic immune response and improves functional recovery after stroke. J. Cereb. Blood Flow Metab..

[97] Hammarlund-Udenaes M., Friden M., Syvanen S., Gupta A. (2008). On the rate and extent of drug delivery to the brain. Pharm. Res..

[98] Pardridge W.M. (2012). Drug transport across the blood-brain barrier. J. Cereb. Blood Flow Metab..

[99] Wong A.D., Ye M., Levy A.F., Rothstein J.D., Bergles D.E., Searson P.C. (2013). The blood-brain barrier: An engineering perspective. Front. Neuroeng..

[100] Ajay, Bemis G.W., Murcko M.A. (1999). Designing libraries with CNS activity. J. Med. Chem..

[101] Fernandez-Garcia L., Mari-Buye N., Barios J.A., Madurga R., Elices M., Perez-Rigueiro J., Ramos M., Guinea G.V., Gonzalez-Nieto D. (2016). Safety and tolerability of silk fibroin hydrogels implanted into the mouse brain. Acta. Biomater..

[102] McCracken P.J., Manduca A., Felmlee J., Ehman R.L. (2005). Mechanical transient-based magnetic resonance elastography. Magn. Reson. Med..

[103] Murphy M.C., Curran G.L., Glaser K.J., Rossman P.J., Huston J., Poduslo J.F., Jack C.R., Felmlee J.P., Ehman R.L. (2012). Magnetic resonance elastography of the brain in a mouse model of Alzheimer’s disease: Initial results. Magn. Reson. Imaging.

[104] Tang-Schomer M.D., White J.D., Tien L.W., Schmitt L.I., Valentin T.M., Graziano D.J., Hopkins A.M., Omenetto F.G., Haydon P.G., Kaplan D.L. (2014). Bioengineered functional brain-like cortical tissue. Proc. Natl. Acad. Sci. USA.

[105] Tang-Schomer M.D., Kaplan D.L., Whalen M.J. (2019). Film interface for drug testing for delivery to cells in culture and in the brain. Acta. Biomater..

[106] Moringlane R.B., Keric N., Freimann F.B., Mielke D., Burger R., Duncker D., Rohde V., Eckardstein K.L.V. (2017). Efficacy and safety of durotomy after decompressive hemicraniectomy in traumatic brain injury. Neurosurg. Rev..

[107] Champagne P.O., Bojanowski M., Fournier-Gosselin M.P., Shedid D. (2018). Safety of performing craniotomy in the elderly: The utility of co-morbidity indices. Interdiscip. Neurosurg..

[108] Baldwin S.A., Fugaccia I., Brown D.R., Brown L.V., Scheff S.W. (1996). Blood-brain barrier breach following cortical contusion in the rat. J. Neurosurg..

[109] Huang Z.G., Xue D., Preston E., Karbalai H., Buchan A.M. (1999). Biphasic opening of the blood-brain barrier following transient focal ischemia: Effects of hypothermia. Can. J. Neurol. Sci..

[110] Hone E.A., Hu H., Sprowls S.A., Farooqi I., Grasmick K., Lockman P.R., Simpkins J.W., Ren X. (2018). Blood-Brain Barrier Openings after Stroke. Neurol. Disord. Stroke Int..

[111] Kuroiwa T., Ting P., Martinez H., Klatzo I. (1985). The biphasic opening of the blood-brain barrier to proteins following temporary middle cerebral artery occlusion. Acta. Neuropathol..

[112] Bharadwaj V.N., Lifshitz J., Adelson P.D., Kodibagkar V.D., Stabenfeldt S.E. (2016). Temporal assessment of nanoparticle accumulation after experimental brain injury: Effect of particle size. Sci. Rep..

[113] Pardridge W.M. (2005). The blood-brain barrier: Bottleneck in brain drug development. NeuroRx.

[114] Mathison S., Nagilla R., Kompella U.B. (1998). Nasal route for direct delivery of solutes to the central nervous system: Fact or fiction?. J. Drug Target..

[115] Kristensson K., Olsson Y. (1971). Uptake of exogenous proteins in mouse olfactory cells. Acta Neuropathol.

[116] Illum L. (2003). Nasal drug delivery--possibilities, problems and solutions. J. Control. Release.

[117] Li R., Huang Y., Chen L., Zhou H., Zhang M., Chang L., Shen H., Zhou M., Su P., Zhu D. (2019). Targeted delivery of intranasally administered nanoparticles-mediated neuroprotective peptide NR2B9c to brain and neuron for treatment of ischemic stroke. Nanomedicine.

[118] Shi J., Kantoff P.W., Wooster R., Farokhzad O.C. (2017). Cancer nanomedicine: Progress, challenges and opportunities. Nat. Rev. Cancer.

[119] Cai W., Chen X. (2007). Nanoplatforms for targeted molecular imaging in living subjects. Small.

[120] Krauze M.T., McKnight T.R., Yamashita Y., Bringas J., Noble C.O., Saito R., Geletneky K., Forsayeth J., Berger M.S., Jackson P. (2005). Real-time visualization and characterization of liposomal delivery into the monkey brain by magnetic resonance imaging. Brain Res. Brain Res. Protoc.

[121] Lee S.R., Lee H.J., Cha S.H., Jeong K.J., Lee Y., Jeon C.Y., Yi K.S., Lim I., Cho Z.H., Chang K.T. (2015). Long-term survival and differentiation of human neural stem cells in nonhuman primate brain with no immunosuppression. Cell Transplant..

[122] Lin B.L., Zhang J.Z., Lu L.J., Mao J.J., Cao M.H., Mao X.H., Zhang F., Duan X.H., Zheng C.S., Zhang L.M. (2017). Superparamagnetic Iron Oxide Nanoparticles-Complexed Cationic Amylose for In Vivo Magnetic Resonance Imaging Tracking of Transplanted Stem Cells in Stroke. Nanomaterials.

[123] Lu Y., Xu Y.J., Zhang G.B., Ling D., Wang M.Q., Zhou Y., Wu Y.D., Wu T., Hackett M.J., Hyo Kim B. (2017). Iron oxide nanoclusters for T 1 magnetic resonance imaging of non-human primates. Nat. Biomed. Eng..

[124] Saito R., Krauze M.T., Bringas J.R., Noble C., McKnight T.R., Jackson P., Wendland M.F., Mamot C., Drummond D.C., Kirpotin D.B. (2005). Gadolinium-loaded liposomes allow for real-time magnetic resonance imaging of convection-enhanced delivery in the primate brain. Exp. Neurol..

[125] Bao G., Mitragotri S., Tong S. (2013). Multifunctional nanoparticles for drug delivery and molecular imaging. Annu Rev. Biomed. Eng.

[126] Feng X., Lv F., Liu L., Tang H., Xing C., Yang Q., Wang S. (2010). Conjugated polymer nanoparticles for drug delivery and imaging. ACS Appl. Mater. Interfaces.

[127] Aktas Y., Yemisci M., Andrieux K., Gursoy R.N., Alonso M.J., Fernandez-Megia E., Novoa-Carballal R., Quinoa E., Riguera R., Sargon M.F. (2005). Development and brain delivery of chitosan-PEG nanoparticles functionalized with the monoclonal antibody OX26. Bioconjugate Chem..

[128] Wohlfart S., Gelperina S., Kreuter J. (2012). Transport of drugs across the blood-brain barrier by nanoparticles. J. Control. Release.

[129] Poellmann M.J., Bu J., Hong S. (2018). Would antioxidant-loaded nanoparticles present an effective treatment for ischemic stroke?. Nanomedicine.

[130] Batrakova E.V., Miller D.W., Li S., Alakhov V.Y., Kabanov A.V., Elmquist W.F. (2001). Pluronic P85 enhances the delivery of digoxin to the brain: In vitro and in vivo studies. J. Pharm. Exp..

[131] Wilson B., Samanta M.K., Santhi K., Kumar K.P., Paramakrishnan N., Suresh B. (2008). Targeted delivery of tacrine into the brain with polysorbate 80-coated poly(n-butylcyanoacrylate) nanoparticles. Eur. J. Pharm. Biopharm..

[132] Wilson B., Samanta M.K., Santhi K., Kumar K.P., Paramakrishnan N., Suresh B. (2008). Poly(n-butylcyanoacrylate) nanoparticles coated with polysorbate 80 for the targeted delivery of rivastigmine into the brain to treat Alzheimer’s disease. Brain Res..

[133] Salvalaio M., Rigon L., Belletti D., D’Avanzo F., Pederzoli F., Ruozi B., Marin O., Vandelli M.A., Forni F., Scarpa M. (2016). Targeted Polymeric Nanoparticles for Brain Delivery of High Molecular Weight Molecules in Lysosomal Storage Disorders. PLoS ONE.

[134] Harris N.M., Ritzel R., Mancini N.S., Jiang Y., Yi X., Manickam D.S., Banks W.A., Kabanov A.V., McCullough L.D., Verma R. (2016). Nano-particle delivery of brain derived neurotrophic factor after focal cerebral ischemia reduces tissue injury and enhances behavioral recovery. Pharmacol. Biochem. Behav..

[135] Kurakhmaeva K.B., Voronina T.A., Kapica I.G., Kreuter J., Nerobkova L.N., Seredenin S.B., Balabanian V.Y., Alyautdin R.N. (2008). Antiparkinsonian effect of nerve growth factor adsorbed on polybutylcyanoacrylate nanoparticles coated with polysorbate-80. Bull. Exp. Biol. Med..

[136] Jeong S.Y., Crooks D.R., Wilson-Ollivierre H., Ghosh M.C., Sougrat R., Lee J., Cooperman S., Mitchell J.B., Beaumont C., Rouault T.A. (2011). Iron insufficiency compromises motor neurons and their mitochondrial function in Irp2-null mice. PLoS ONE.

[137] Lu W.L., Qi X.R., Zhang Q., Li R.Y., Wang G.L., Zhang R.J., Wei S.L. (2004). A pegylated liposomal platform: Pharmacokinetics, pharmacodynamics, and toxicity in mice using doxorubicin as a model drug. J. Pharmacol. Sci..

[138] Tiwari S.B., Amiji M.M. (2006). A review of nanocarrier-based CNS delivery systems. Curr. Drug Deliv..

[139] Hong H.Y., Choi J.S., Kim Y.J., Lee H.Y., Kwak W., Yoo J., Lee J.T., Kwon T.H., Kim I.S., Han H.S. (2008). Detection of apoptosis in a rat model of focal cerebral ischemia using a homing peptide selected from in vivo phage display. J. Control. Release.

[140] Lv W., Xu J., Wang X., Li X., Xu Q., Xin H. (2018). Bioengineered Boronic Ester Modified Dextran Polymer Nanoparticles as Reactive Oxygen Species Responsive Nanocarrier for Ischemic Stroke Treatment. ACS Nano.

[141] Lockman P.R., Koziara J.M., Mumper R.J., Allen D.D. (2004). Nanoparticle surface charges alter blood-brain barrier integrity and permeability. J. Drug Target..

[142] Bharadwaj V.N., Nguyen D.T., Kodibagkar V.D., Stabenfeldt S.E. (2018). Nanoparticle-Based Therapeutics for Brain Injury. Adv. Healthc Mater..

[143] Davis M.E., Chen Z.G., Shin D.M. (2008). Nanoparticle therapeutics: An emerging treatment modality for cancer. Nat. Rev. Drug Discov..

[144] Lai X., Zhao H., Zhang Y., Guo K., Xu Y., Chen S., Zhang J. (2018). Intranasal Delivery of Copper Oxide Nanoparticles Induces Pulmonary Toxicity and Fibrosis in C57BL/6 mice. Sci. Rep..

[145] Santos S.D., Xavier M., Leite D.M., Moreira D.A., Custodio B., Torrado M., Castro R., Leiro V., Rodrigues J., Tomas H. (2018). PAMAM dendrimers: Blood-brain barrier transport and neuronal uptake after focal brain ischemia. J. Control. Release.

[146] Song Q., Song H., Xu J., Huang J., Hu M., Gu X., Chen J., Zheng G., Chen H., Gao X. (2016). Biomimetic ApoE-Reconstituted High Density Lipoprotein Nanocarrier for Blood-Brain Barrier Penetration and Amyloid Beta-Targeting Drug Delivery. Mol. Pharm..

[147] Godinho B., Henninger N., Bouley J., Alterman J.F., Haraszti R.A., Gilbert J.W., Sapp E., Coles A.H., Biscans A., Nikan M. (2018). Transvascular Delivery of Hydrophobically Modified siRNAs: Gene Silencing in the Rat Brain upon Disruption of the Blood-Brain Barrier. Mol. Ther..

[148] Dos Santos Rodrigues B., Oue H., Banerjee A., Kanekiyo T., Singh J. (2018). Dual functionalized liposome-mediated gene delivery across triple co-culture blood brain barrier model and specific in vivo neuronal transfection. J. Control. Release.

[149] Xing Y., Wen C.Y., Li S.T., Xia Z.X. (2016). Non-viral liposome-mediated transfer of brain-derived neurotrophic factor across the blood-brain barrier. Neural Regen Res..

[150] Nour S.A., Abdelmalak N.S., Naguib M.J., Rashed H.M., Ibrahim A.B. (2016). Intranasal brain-targeted clonazepam polymeric micelles for immediate control of status epilepticus: In vitro optimization, ex vivo determination of cytotoxicity, in vivo biodistribution and pharmacodynamics studies. Drug Deliv..

[151] Basalious E.B., Shamma R.N. (2015). Novel self-assembled nano-tubular mixed micelles of Pluronics P123, Pluronic F127 and phosphatidylcholine for oral delivery of nimodipine: In vitro characterization, ex vivo transport and in vivo pharmacokinetic studies. Int. J. Pharm..

[152] Jin Q., Cai Y., Li S., Liu H., Zhou X., Lu C., Gao X., Qian J., Zhang J., Ju S. (2017). Edaravone-Encapsulated Agonistic Micelles Rescue Ischemic Brain Tissue by Tuning Blood-Brain Barrier Permeability. Theranostics.

[153] Tuladhar A., Morshead C.M., Shoichet M.S. (2015). Circumventing the blood-brain barrier: Local delivery of cyclosporin A stimulates stem cells in stroke-injured rat brain. J. Control. Release.

[154] Wang Y., Cooke M.J., Sachewsky N., Morshead C.M., Shoichet M.S. (2013). Bioengineered sequential growth factor delivery stimulates brain tissue regeneration after stroke. J. Control. Release.

[155] Bjugstad K.B., Redmond D.E., Lampe K.J., Kern D.S., Sladek J.R., Mahoney M.J. (2008). Biocompatibility of PEG-based hydrogels in primate brain. Cell Transplant..

[156] Cook D.J., Nguyen C., Chun H.N., Irene L.L., Chiu A.S., Machnicki M., Zarembinski T.I., Carmichael S.T. (2017). Hydrogel-delivered brain-derived neurotrophic factor promotes tissue repair and recovery after stroke. J. Cereb. Blood Flow Metab..

[157] Murphy M.C., Jones D.T., Jack C.R., Glaser K.J., Senjem M.L., Manduca A., Felmlee J.P., Carter R.E., Ehman R.L., Huston J. (2016). Regional brain stiffness changes across the Alzheimer’s disease spectrum. Neuroimage Clin..

[158] Chen X., Zhi F., Jia X., Zhang X., Ambardekar R., Meng Z., Paradkar A.R., Hu Y., Yang Y. (2013). Enhanced brain targeting of curcumin by intranasal administration of a thermosensitive poloxamer hydrogel. J. Pharm Pharmacol..

[159] Hoban D.B., Newland B., Moloney T.C., Howard L., Pandit A., Dowd E. (2013). The reduction in immunogenicity of neurotrophin overexpressing stem cells after intra-striatal transplantation by encapsulation in an in situ gelling collagen hydrogel. Biomaterials.

[160] Ballios B.G., Cooke M.J., Donaldson L., Coles B.L., Morshead C.M., van der Kooy D., Shoichet M.S. (2015). A Hyaluronan-Based Injectable Hydrogel Improves the Survival and Integration of Stem Cell Progeny following Transplantation. Stem Cell Rep..

[161] Nih L.R., Sideris E., Carmichael S.T., Segura T. (2017). Injection of Microporous Annealing Particle (MAP) Hydrogels in the Stroke Cavity Reduces Gliosis and Inflammation and Promotes NPC Migration to the Lesion. Adv. Mater..

[162] Caicco M.J., Cooke M.J., Wang Y., Tuladhar A., Morshead C.M., Shoichet M.S. (2013). A hydrogel composite system for sustained epi-cortical delivery of Cyclosporin A to the brain for treatment of stroke. J. Control. Release.

[163] Osanai T., Kuroda S., Yasuda H., Chiba Y., Maruichi K., Hokari M., Sugiyama T., Shichinohe H., Iwasaki Y. (2010). Noninvasive transplantation of bone marrow stromal cells for ischemic stroke: Preliminary study with a thermoreversible gelation polymer hydrogel. Neurosurgery.

[164] Mun E.A., Hannell C., Rogers S.E., Hole P., Williams A.C., Khutoryanskiy V.V. (2014). On the role of specific interactions in the diffusion of nanoparticles in aqueous polymer solutions. Langmuir.

[165] Nance E.A., Woodworth G.F., Sailor K.A., Shih T.Y., Xu Q., Swaminathan G., Xiang D., Eberhart C., Hanes J. (2012). A dense poly(ethylene glycol) coating improves penetration of large polymeric nanoparticles within brain tissue. Sci. Transl. Med..

[166] Cruz L.J., Stammes M.A., Que I., van Beek E.R., Knol-Blankevoort V.T., Snoeks T.J.A., Chan A., Kaijzel E.L., Lowik C. (2016). Effect of PLGA NP size on efficiency to target traumatic brain injury. J. Control. Release.

[167] Potjewyd G., Moxon S., Wang T., Domingos M., Hooper N.M. (2018). Tissue Engineering 3D Neurovascular Units: A Biomaterials and Bioprinting Perspective. Trends Biotechnol..

[168] Emerich D.F., Silva E., Ali O., Mooney D., Bell W., Yu S.J., Kaneko Y., Borlongan C. (2010). Injectable VEGF hydrogels produce near complete neurological and anatomical protection following cerebral ischemia in rats. Cell Transplant..

[169] Hao P., Duan H., Hao F., Chen L., Sun M., Fan K.S., Sun Y.E., Williams D., Yang Z., Li X. (2017). Neural repair by NT3-chitosan via enhancement of endogenous neurogenesis after adult focal aspiration brain injury. Biomaterials.

[170] Obermeyer J.M., Tuladhar A., Payne S.L., Ho E., Morshead C.M., Shoichet M.S. (2019). Local Delivery of Brain-Derived Neurotrophic Factor Enables Behavioral Recovery and Tissue Repair in Stroke-Injured Rats. Tissue Eng. Part. A.

[171] Overman J.J., Clarkson A.N., Wanner I.B., Overman W.T., Eckstein I., Maguire J.L., Dinov I.D., Toga A.W., Carmichael S.T. (2012). A role for ephrin-A5 in axonal sprouting, recovery, and activity-dependent plasticity after stroke. Proc. Natl. Acad. Sci. USA.

[172] Tian W.M., Zhang C.L., Hou S.P., Yu X., Cui F.Z., Xu Q.Y., Sheng S.L., Cui H., Li H.D. (2005). Hyaluronic acid hydrogel as Nogo-66 receptor antibody delivery system for the repairing of injured rat brain: In vitro. J. Control. Release.

[173] Martin-Martin Y., Fernandez-Garcia L., Sanchez-Rebato M.H., Mari-Buye N., Rojo F.J., Perez-Rigueiro J., Ramos M., Guinea G.V., Panetsos F., Gonzalez-Nieto D. (2019). Evaluation of Neurosecretome from Mesenchymal Stem Cells Encapsulated in Silk Fibroin Hydrogels. Sci Rep..

[174] Palejwala A.H., Fridley J.S., Mata J.A., Samuel E.L., Luerssen T.G., Perlaky L., Kent T.A., Tour J.M., Jea A. (2016). Biocompatibility of reduced graphene oxide nanoscaffolds following acute spinal cord injury in rats. Surg. Neurol. Int..

[175] Lampe K.J., Mooney R.G., Bjugstad K.B., Mahoney M.J. (2010). Effect of macromer weight percent on neural cell growth in 2D and 3D nondegradable PEG hydrogel culture. J. Biomed. Mater. Res. A.

[176] Struzyna L.A., Wolf J.A., Mietus C.J., Adewole D.O., Chen H.I., Smith D.H., Cullen D.K. (2015). Rebuilding Brain Circuitry with Living Micro-Tissue Engineered Neural Networks. Tissue Eng. Part. A.

[177] Kloxin A.M., Kasko A.M., Salinas C.N., Anseth K.S. (2009). Photodegradable hydrogels for dynamic tuning of physical and chemical properties. Science.

[178] Galarraga J.H., Burdick J.A. (2019). Moving hydrogels to the fourth dimension. Nat. Mater..

[179] Shadish J.A., Benuska G.M., DeForest C.A. (2019). Bioactive site-specifically modified proteins for 4D patterning of gel biomaterials. Nat. Mater..

[180] Adak A., Das G., Khan J., Mukherjee N., Gupta V., Mallesh R., Ghosh S. (2020). Extracellular Matrix Mimicking (ECM) Neuroprotective Injectable Sulfo-functionalized Peptide Hydrogel for Repairing Brain Injury. ACS Biomater. Sci. Eng..

[181] Ghuman H., Massensini A.R., Donnelly J., Kim S.M., Medberry C.J., Badylak S.F., Modo M. (2016). ECM hydrogel for the treatment of stroke: Characterization of the host cell infiltrate. Biomaterials.

[182] Ghuman H., Mauney C., Donnelly J., Massensini A.R., Badylak S.F., Modo M. (2018). Biodegradation of ECM hydrogel promotes endogenous brain tissue restoration in a rat model of stroke. Acta. Biomater..

[183] Fabian R.H., Derry P.J., Rea H.C., Dalmeida W.V., Nilewski L.G., Sikkema W.K.A., Mandava P., Tsai A.L., Mendoza K., Berka V. (2018). Efficacy of Novel Carbon Nanoparticle Antioxidant Therapy in a Severe Model of Reversible Middle Cerebral Artery Stroke in Acutely Hyperglycemic Rats. Front. Neurol..

[184] Amani H., Habibey R., Shokri F., Hajmiresmail S.J., Akhavan O., Mashaghi A., Pazoki-Toroudi H. (2019). Selenium nanoparticles for targeted stroke therapy through modulation of inflammatory and metabolic signaling. Sci. Rep..

[185] Korin N., Kanapathipillai M., Matthews B.D., Crescente M., Brill A., Mammoto T., Ghosh K., Jurek S., Bencherif S.A., Bhatta D. (2012). Shear-activated nanotherapeutics for drug targeting to obstructed blood vessels. Science.

[186] Sweeney M.D., Ayyadurai S., Zlokovic B.V. (2016). Pericytes of the neurovascular unit: Key functions and signaling pathways. Nat. Neurosci..

[187] Kuo Y.C., Lu C.H. (2011). Effect of human astrocytes on the characteristics of human brain-microvascular endothelial cells in the blood-brain barrier. Colloids Surf. B Biointerfaces.

[188] Thored P., Wood J., Arvidsson A., Cammenga J., Kokaia Z., Lindvall O. (2007). Long-term neuroblast migration along blood vessels in an area with transient angiogenesis and increased vascularization after stroke. Stroke.

[189] Ohab J.J., Fleming S., Blesch A., Carmichael S.T. (2006). A neurovascular niche for neurogenesis after stroke. J. Neurosci..

[190] Ali Z., Islam A., Sherrell P., Le-Moine M., Lolas G., Syrigos K., Rafat M., Jensen L.D. (2018). Adjustable delivery of pro-angiogenic FGF-2 by alginate:collagen microspheres. Biol. Open.

[191] Bible E., Qutachi O., Chau D.Y., Alexander M.R., Shakesheff K.M., Modo M. (2012). Neo-vascularization of the stroke cavity by implantation of human neural stem cells on VEGF-releasing PLGA microparticles. Biomaterials.

[192] Ju R., Wen Y., Gou R., Wang Y., Xu Q. (2014). The experimental therapy on brain ischemia by improvement of local angiogenesis with tissue engineering in the mouse. Cell Transplant..

[193] Tan E.Y., Law J.W., Wang C.H., Lee A.Y. (2007). Development of a cell transducible RhoA inhibitor TAT-C3 transferase and its encapsulation in biocompatible microspheres to promote survival and enhance regeneration of severed neurons. Pharm. Res..

[194] Deguchi K., Tsuru K., Hayashi T., Takaishi M., Nagahara M., Nagotani S., Sehara Y., Jin G., Zhang H., Hayakawa S. (2006). Implantation of a new porous gelatin-siloxane hybrid into a brain lesion as a potential scaffold for tissue regeneration. J. Cereb. Blood Flow Metab..

[195] Yang Z., Zhang A., Duan H., Zhang S., Hao P., Ye K., Sun Y.E., Li X. (2015). NT3-chitosan elicits robust endogenous neurogenesis to enable functional recovery after spinal cord injury. Proc. Natl. Acad. Sci. USA.

[196] Chen H., Spagnoli F., Burris M., Rolland W.B., Fajilan A., Dou H., Tang J., Zhang J.H. (2012). Nanoerythropoietin is 10-times more effective than regular erythropoietin in neuroprotection in a neonatal rat model of hypoxia and ischemia. Stroke.

[197] Elizarova O.S., Litvinova S.A., Balaban’ian V., Barskov I.V., Novikova S.V., Stel’mashuk E.V., Garibova T.L., Voronina T.A. (2012). Neuroprotective effect of recombinant human erythropoietin-loaded poly(lactic-co-glycolic) acid nanoparticles in rats with intracerebral posttraumatic hematoma. Eksp Klin Farm..

[198] Solev I.N., Balabanyan V.Y., Volchek I.A., Elizarova O.S., Litvinova S.A., Garibova T.L., Voronina T.A. (2013). Involvement of BDNF and NGF in the mechanism of neuroprotective effect of human recombinant erythropoietin nanoforms. Bull. Exp. Biol. Med..

[199] Jin Y., Kim I.Y., Kim I.D., Lee H.K., Park J.Y., Han P.L., Kim K.K., Choi H., Lee J.K. (2014). Biodegradable gelatin microspheres enhance the neuroprotective potency of osteopontin via quick and sustained release in the post-ischemic brain. Acta. Biomater..

[200] Joachim E., Kim I.D., Jin Y., Kim K.K., Lee J.K., Choi H. (2014). Gelatin nanoparticles enhance the neuroprotective effects of intranasally administered osteopontin in rat ischemic stroke model. Drug Deliv. Transl. Res..

[201] Navath R.S., Kurtoglu Y.E., Wang B., Kannan S., Romero R., Kannan R.M. (2008). Dendrimer-drug conjugates for tailored intracellular drug release based on glutathione levels. Bioconjug. Chem..

[202] Butterfield D.A., Halliwell B. (2019). Oxidative stress, dysfunctional glucose metabolism and Alzheimer disease. Nat. Rev. Neurosci..

[203] Reddy M.K., Labhasetwar V. (2009). Nanoparticle-mediated delivery of superoxide dismutase to the brain: An effective strategy to reduce ischemia-reperfusion injury. FASEB J..

[204] Singhal A., Morris V.B., Labhasetwar V., Ghorpade A. (2013). Nanoparticle-mediated catalase delivery protects human neurons from oxidative stress. Cell Death Dis..

[205] Jiang Y., Brynskikh A.M., Devika S., Kabanov A.V. (2015). SOD1 nanozyme salvages ischemic brain by locally protecting cerebral vasculature. J. Control. Release.

[206] Manickam D.S., Brynskikh A.M., Kopanic J.L., Sorgen P.L., Klyachko N.L., Batrakova E.V., Bronich T.K., Kabanov A.V. (2012). Well-defined cross-linked antioxidant nanozymes for treatment of ischemic brain injury. J. Control. Release.

[207] Alkharfy K.M., Ahmad A., Khan R.M., Al-Shagha W.M. (2015). Pharmacokinetic plasma behaviors of intravenous and oral bioavailability of thymoquinone in a rabbit model. Eur. J. Drug Metab. Pharmacokinet..

[208] Xiao X.Y., Zhu Y.X., Bu J.Y., Li G.W., Zhou J.H., Zhou S.P. (2016). Evaluation of Neuroprotective Effect of Thymoquinone Nanoformulation in the Rodent Cerebral Ischemia-Reperfusion Model. Biomed. Res. Int..

[209] Haque S., Md S., Fazil M., Kumar M., Sahni J.K., Ali J., Baboota S. (2012). Venlafaxine loaded chitosan NPs for brain targeting: Pharmacokinetic and pharmacodynamic evaluation. Carbohydr. Polym..

[210] Ding Y., Qiao Y., Wang M., Zhang H., Li L., Zhang Y., Ge J., Song Y., Li Y., Wen A. (2016). Enhanced Neuroprotection of Acetyl-11-Keto-beta-Boswellic Acid (AKBA)-Loaded O-Carboxymethyl Chitosan Nanoparticles Through Antioxidant and Anti-Inflammatory Pathways. Mol. Neurobiol..

[211] Klose D., Laprais M., Leroux V., Siepmann F., Deprez B., Bordet R., Siepmann J. (2009). Fenofibrate-loaded PLGA microparticles: Effects on ischemic stroke. Eur. J. Pharm. Sci..

[212] Floyd R.A., Kopke R.D., Choi C.H., Foster S.B., Doblas S., Towner R.A. (2008). Nitrones as therapeutics. Free Radic. Biol. Med..

[213] Pinarbasli O., Aktas Y., Dalkara T., Andrieux K., Alonso M.J., Fernandez-Megia E., Novoa-Carballal R., Riguera R., Couvreur P., Capan Y. (2009). Preparation and evaluation of alpha-phenyl-n-tert-butyl nitrone (PBN)-encapsulated chitosan and PEGylated chitosan nanoparticles. Pharmazie.

[214] Hazekawa M., Sakai Y., Yoshida M., Haraguchi T., Uchida T. (2012). Single injection of ONO-1301-loaded PLGA microspheres directly after ischaemia reduces ischaemic damage in rats subjected to middle cerebral artery occlusion. J. Pharm. Pharmacol..

[215] Kakkar V., Muppu S.K., Chopra K., Kaur I.P. (2013). Curcumin loaded solid lipid nanoparticles: An efficient formulation approach for cerebral ischemic reperfusion injury in rats. Eur. J. Pharm. Biopharm..

[216] Zhao Y., Jiang Y., Lv W., Wang Z., Lv L., Wang B., Liu X., Liu Y., Hu Q., Sun W. (2016). Dual targeted nanocarrier for brain ischemic stroke treatment. J. Control. Release.

[217] Chaturvedi M., Figiel I., Sreedhar B., Kaczmarek L. (2012). Neuroprotection from tissue inhibitor of metalloproteinase-1 and its nanoparticles. Neurochem. Int..

[218] Chaturvedi M., Molino Y., Sreedhar B., Khrestchatisky M., Kaczmarek L. (2014). Tissue inhibitor of matrix metalloproteinases-1 loaded poly(lactic-co-glycolic acid) nanoparticles for delivery across the blood-brain barrier. Int. J. Nanomed..

[219] Verma S.K., Arora I., Javed K., Akhtar M., Samim M. (2016). Enhancement in the Neuroprotective Power of Riluzole Against Cerebral Ischemia Using a Brain Targeted Drug Delivery Vehicle. ACS Appl. Mater. Interfaces.

[220] Wahl A.S., Omlor W., Rubio J.C., Chen J.L., Zheng H., Schroter A., Gullo M., Weinmann O., Kobayashi K., Helmchen F. (2014). Neuronal repair. Asynchronous therapy restores motor control by rewiring of the rat corticospinal tract after stroke. Science.

[221] Schwab M.E. (2010). Functions of Nogo proteins and their receptors in the nervous system. Nat. Rev. Neurosci..

[222] Han L., Cai Q., Tian D., Kong D.K., Gou X., Chen Z., Strittmatter S.M., Wang Z., Sheth K.N., Zhou J. (2016). Targeted drug delivery to ischemic stroke via chlorotoxin-anchored, lexiscan-loaded nanoparticles. Nanomedicine.

[223] Nagai N., Yoshioka C., Ito Y., Funakami Y., Nishikawa H., Kawabata A. (2015). Intravenous Administration of Cilostazol Nanoparticles Ameliorates Acute Ischemic Stroke in a Cerebral Ischemia/Reperfusion-Induced Injury Model. Int. J. Mol. Sci..

[224] Chen C., Mei H., Shi W., Deng J., Zhang B., Guo T., Wang H., Hu Y. (2013). EGFP-EGF1-conjugated PLGA nanoparticles for targeted delivery of siRNA into injured brain microvascular endothelial cells for efficient RNA interference. PLoS ONE.

[225] Lee J., Hyun H., Kim J., Ryu J.H., Kim H.A., Park J.H., Lee M. (2012). Dexamethasone-loaded peptide micelles for delivery of the heme oxygenase-1 gene to ischemic brain. J. Control. Release.

[226] Kim I.D., Lim C.M., Kim J.B., Nam H.Y., Nam K., Kim S.W., Park J.S., Lee J.K. (2010). Neuroprotection by biodegradable PAMAM ester (e-PAM-R)-mediated HMGB1 siRNA delivery in primary cortical cultures and in the postischemic brain. J. Control. Release.

[227] Wang C., Lin G., Luan Y., Ding J., Li P.C., Zhao Z., Qian C., Liu G., Ju S., Teng G.J. (2019). HIF-prolyl hydroxylase 2 silencing using siRNA delivered by MRI-visible nanoparticles improves therapy efficacy of transplanted EPCs for ischemic stroke. Biomaterials.

[228] Gonzalez-Nieto D., Li L., Kohler A., Ghiaur G., Ishikawa E., Sengupta A., Madhu M., Arnett J.L., Santho R.A., Dunn S.K. (2012). Connexin-43 in the osteogenic BM niche regulates its cellular composition and the bidirectional traffic of hematopoietic stem cells and progenitors. Blood.

[229] Kim D.H., Seo Y.K., Thambi T., Moon G.J., Son J.P., Li G., Park J.H., Lee J.H., Kim H.H., Lee D.S. (2015). Enhancing neurogenesis and angiogenesis with target delivery of stromal cell derived factor-1alpha using a dual ionic pH-sensitive copolymer. Biomaterials.

[230] Boisserand L.S., Kodama T., Papassin J., Auzely R., Moisan A., Rome C., Detante O. (2016). Biomaterial Applications in Cell-Based Therapy in Experimental Stroke. Stem Cells Int..

[231] Gonzalez-Nieto D., Fernández-García L., Pérez-Rigueiro J., Guinea G.V., Panetsos F. (2018). Hydrogels-Assisted Cell Engraftment for Repairing the Stroke-Damaged Brain: Chimera or Reality. Polymers.

[232] Hwang B.W., Kim S.J., Park K.M., Kim H., Yeom J., Yang J.A., Jeong H., Jung H., Kim K., Sung Y.C. (2015). Genetically engineered mesenchymal stem cell therapy using self-assembling supramolecular hydrogels. J. Control. Release.

[233] Nih L.R., Carmichael S.T., Segura T. (2016). Hydrogels for brain repair after stroke: An emerging treatment option. Curr. Opin. Biotechnol..

[234] Shi M., Sanche L. (2019). Convection-Enhanced Delivery in Malignant Gliomas: A Review of Toxicity and Efficacy. J. Oncol..

[235] Kawaguchi A.T., Haida M., Ohba H., Yamano M., Fukumoto D., Tsukada H. (2013). Liposome-encapsulated hemoglobin ameliorates ischemic stroke in nonhuman primates: Longitudinal observation. Artif. Organs.

[236] Kawaguchi A.T., Haida M., Yamano M., Fukumoto D., Ogata Y., Tsukada H. (2010). Liposome-encapsulated hemoglobin ameliorates ischemic stroke in nonhuman primates: An acute study. J. Pharmacol. Exp. Ther..

